# Supplementary figures and images for: Exploring the expression patterns of palmitoylating and de-palmitoylating enzymes in the mouse brain using the curated RNA-seq database BrainPalmSeq
Source: eLife. 2022 Jul 12;11:e75804. doi: 10.7554/eLife.75804 (PMC9365392; doi:10.7554/eLife.75804)

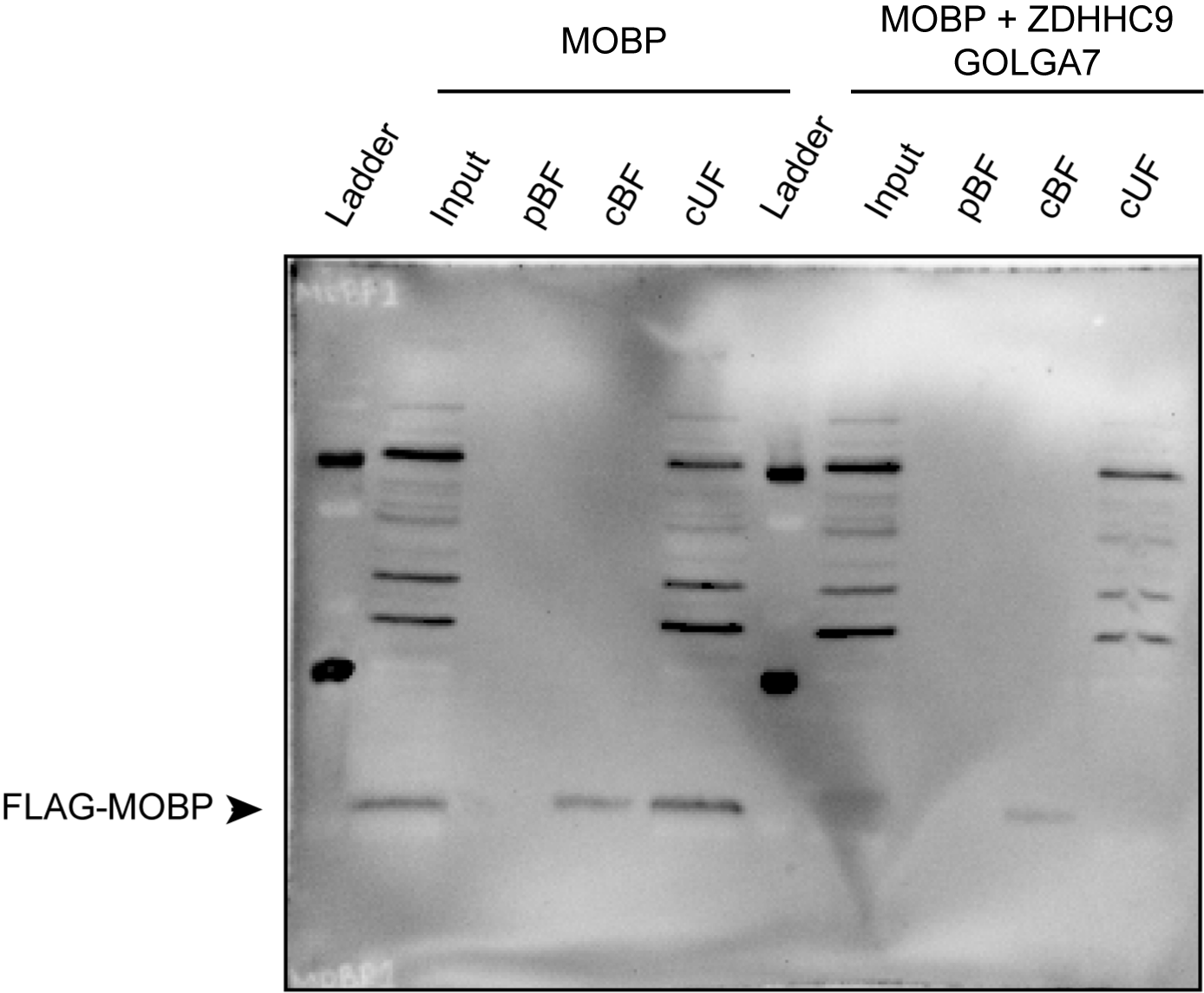

Supplement: Figure 6—source data 4. [file elife-75804-fig6-data4.zip › Figure 6 - Source data 10.tif]

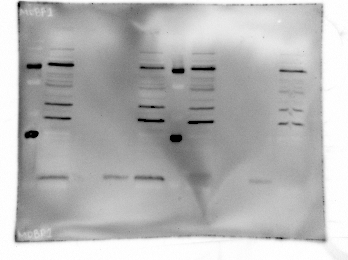

Supplement: Figure 6—source data 4. [file elife-75804-fig6-data4.zip › Figure 6 - Source data 11.tif]

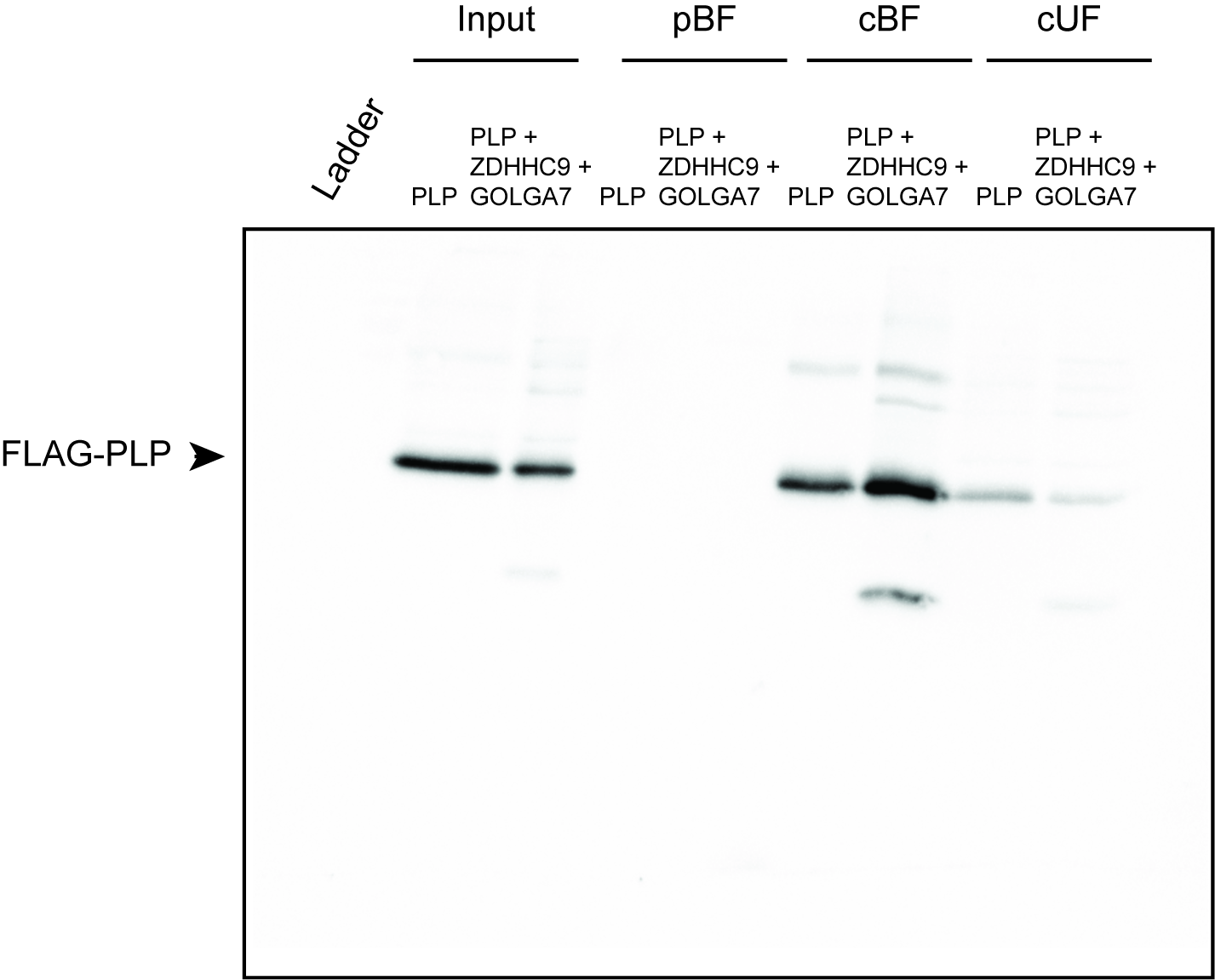

Supplement: Figure 6—source data 4. [file elife-75804-fig6-data4.zip › Figure 6 - Source data 12.tif]

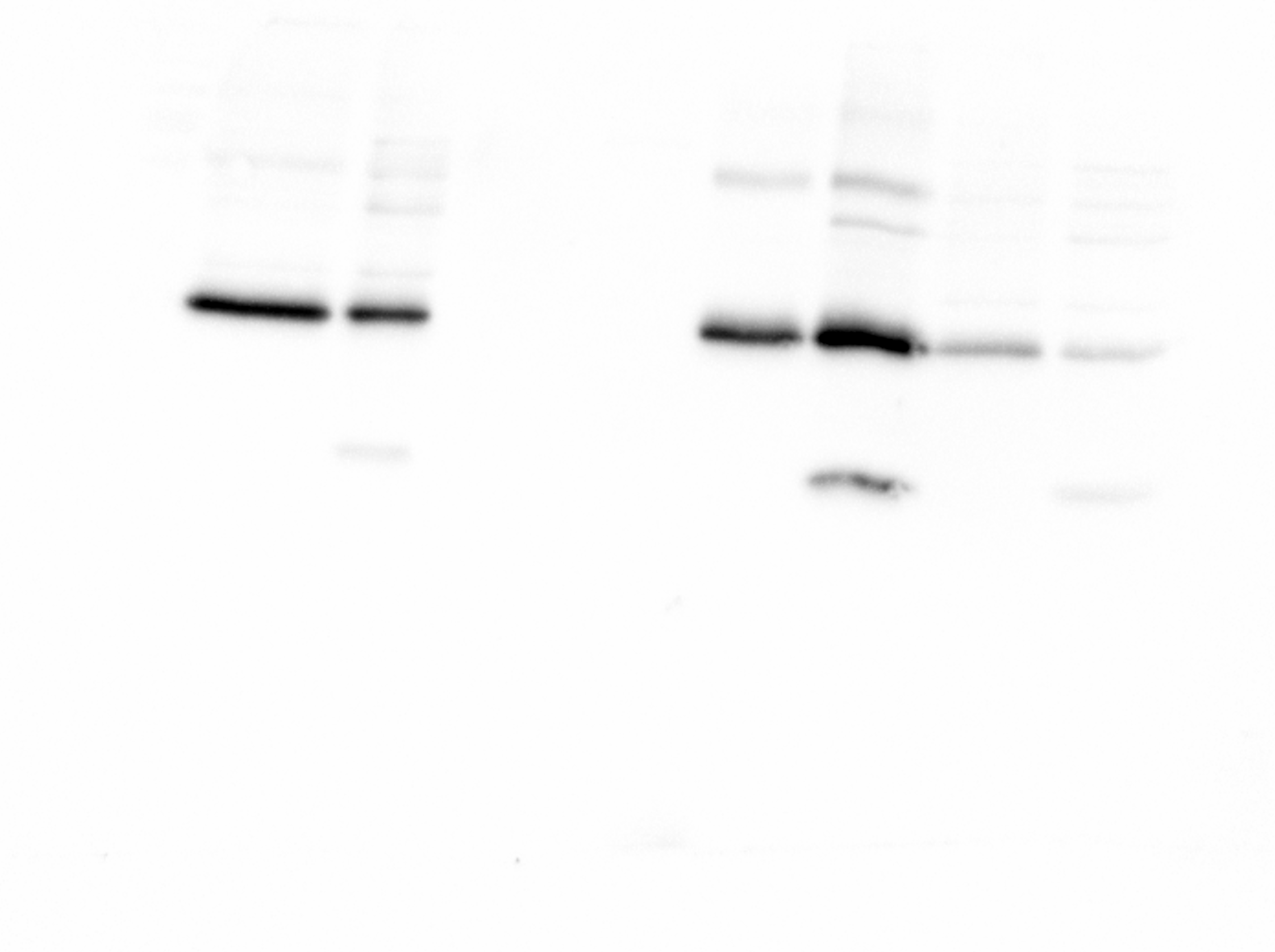

Supplement: Figure 6—source data 4. [file elife-75804-fig6-data4.zip › Figure 6 - Source data 13.tif]

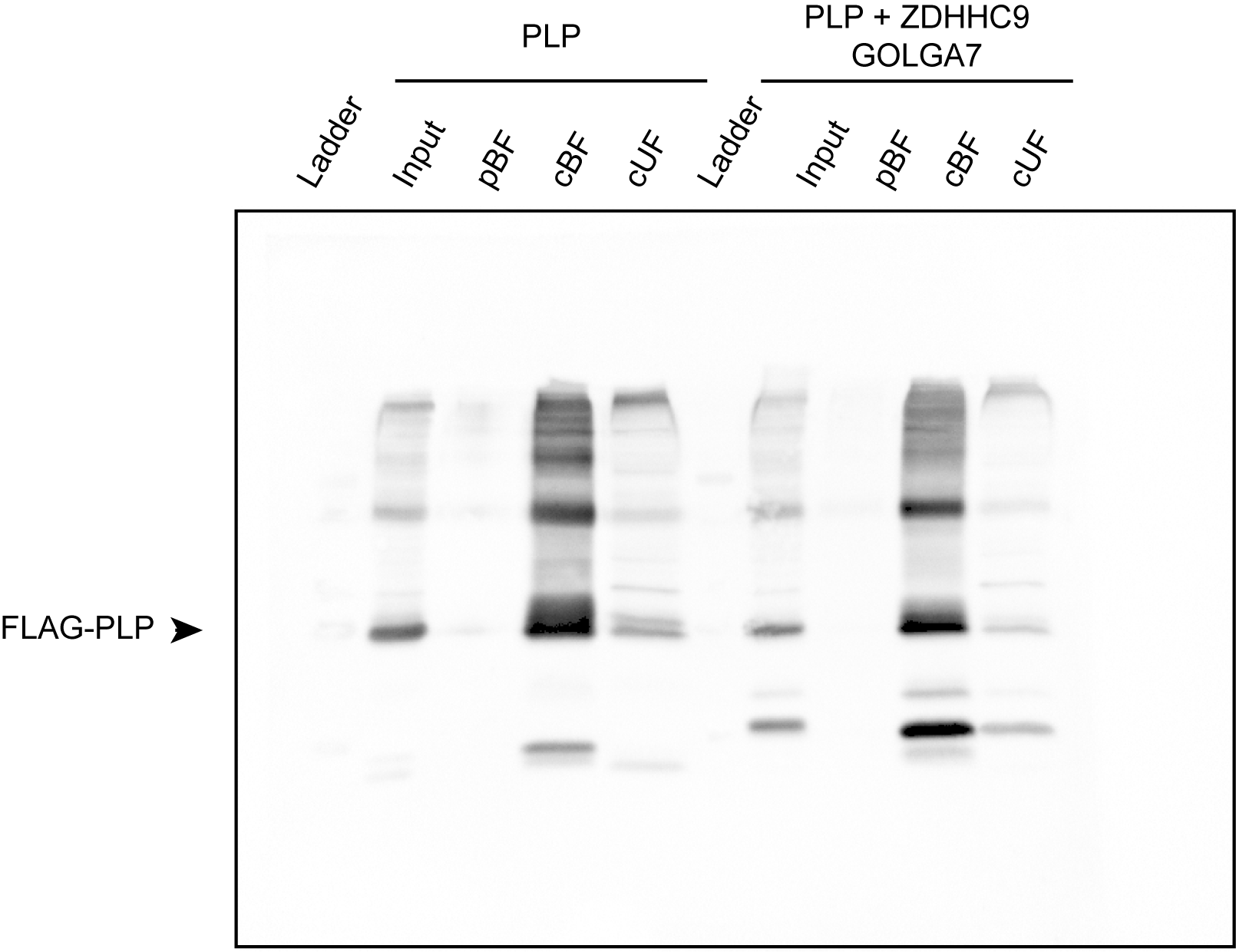

Supplement: Figure 6—source data 4. [file elife-75804-fig6-data4.zip › Figure 6 - Source data 14.tif]

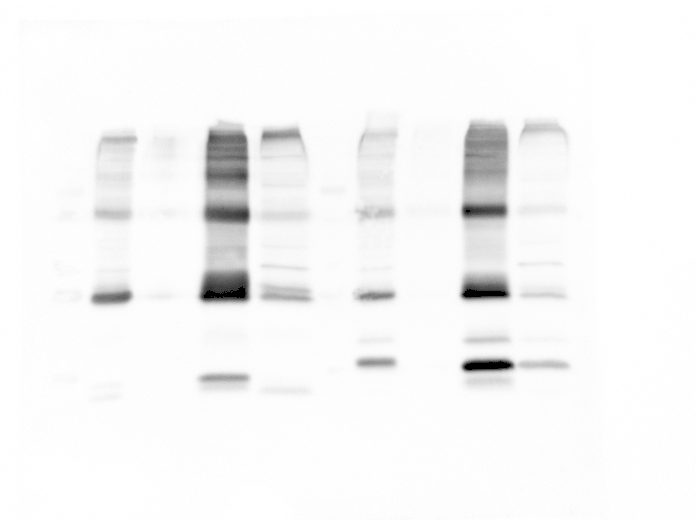

Supplement: Figure 6—source data 4. [file elife-75804-fig6-data4.zip › Figure 6 - Source data 15.tif]

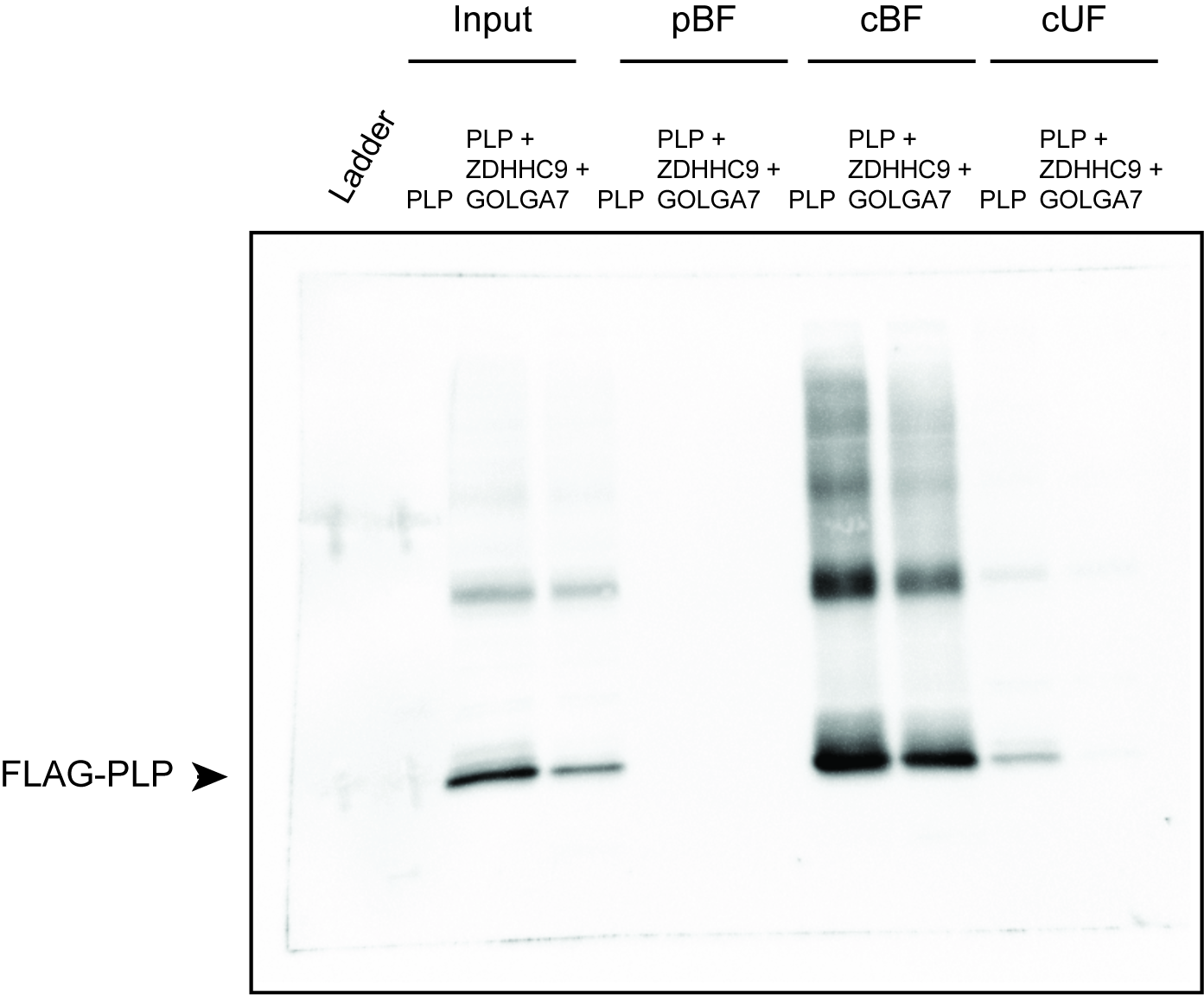

Supplement: Figure 6—source data 4. [file elife-75804-fig6-data4.zip › Figure 6 - Source data 16.tif]

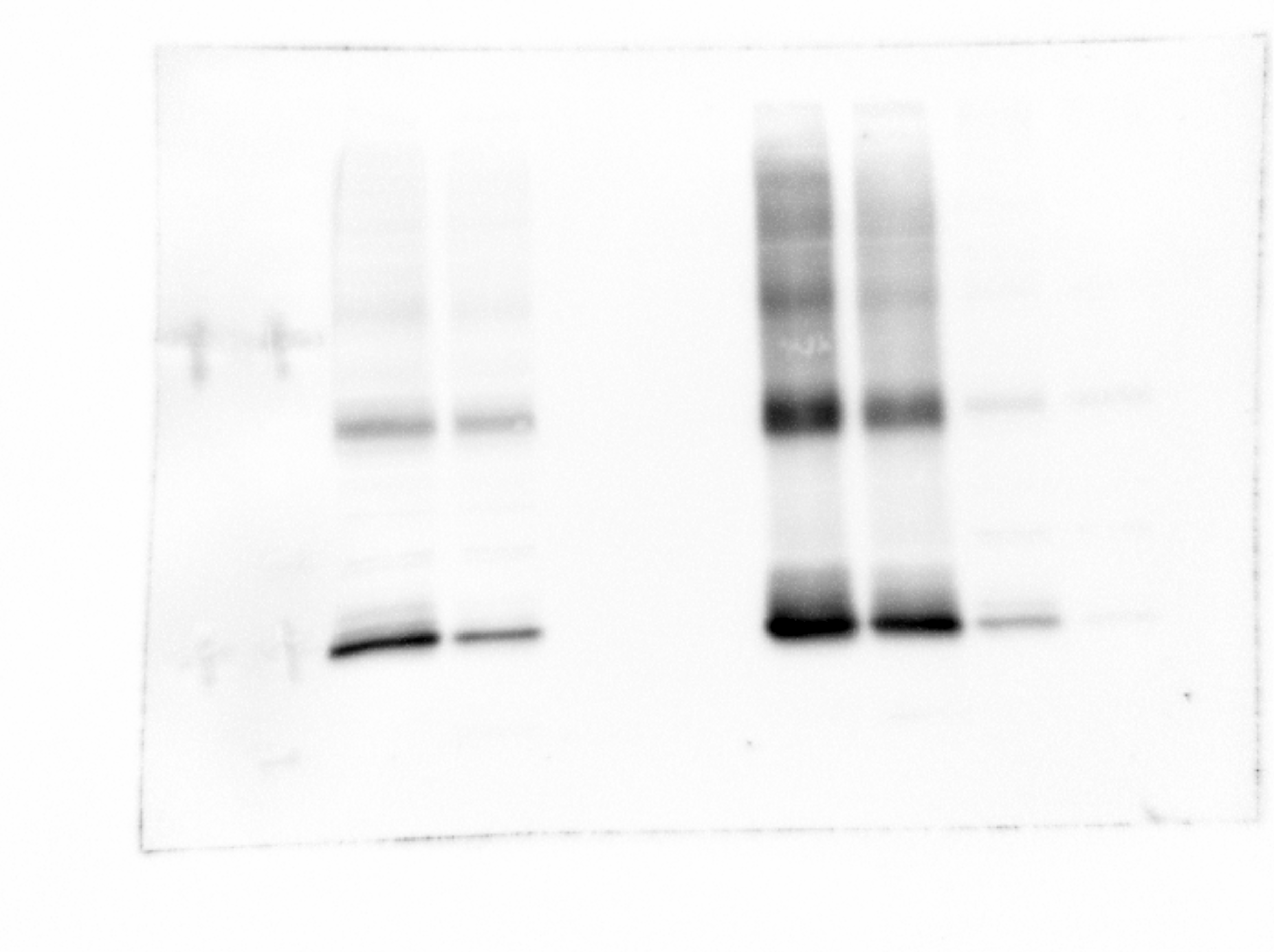

Supplement: Figure 6—source data 4. [file elife-75804-fig6-data4.zip › Figure 6 - Source data 17.tif]

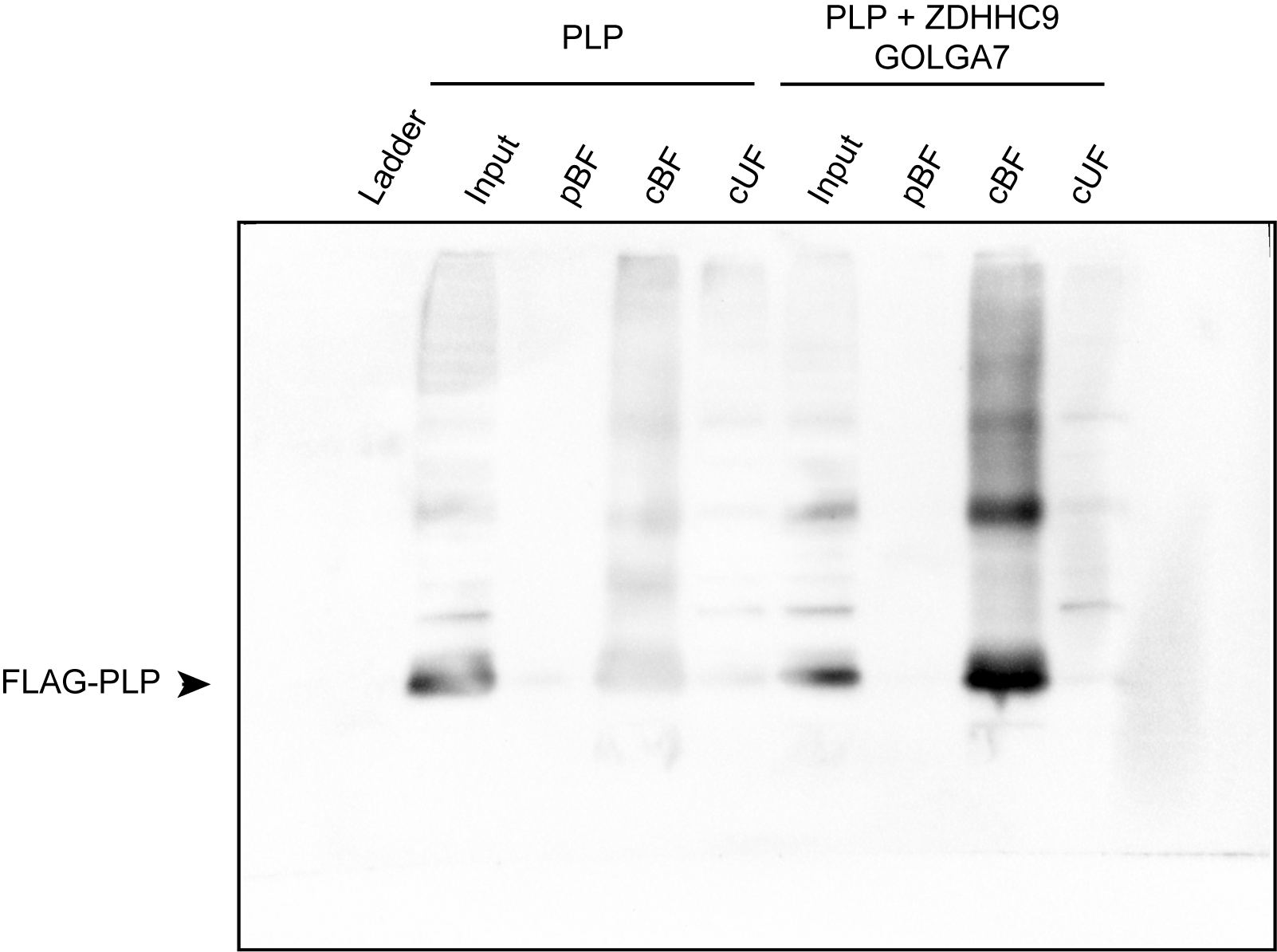

Supplement: Figure 6—source data 4. [file elife-75804-fig6-data4.zip › Figure 6 - Source data 18.tif]

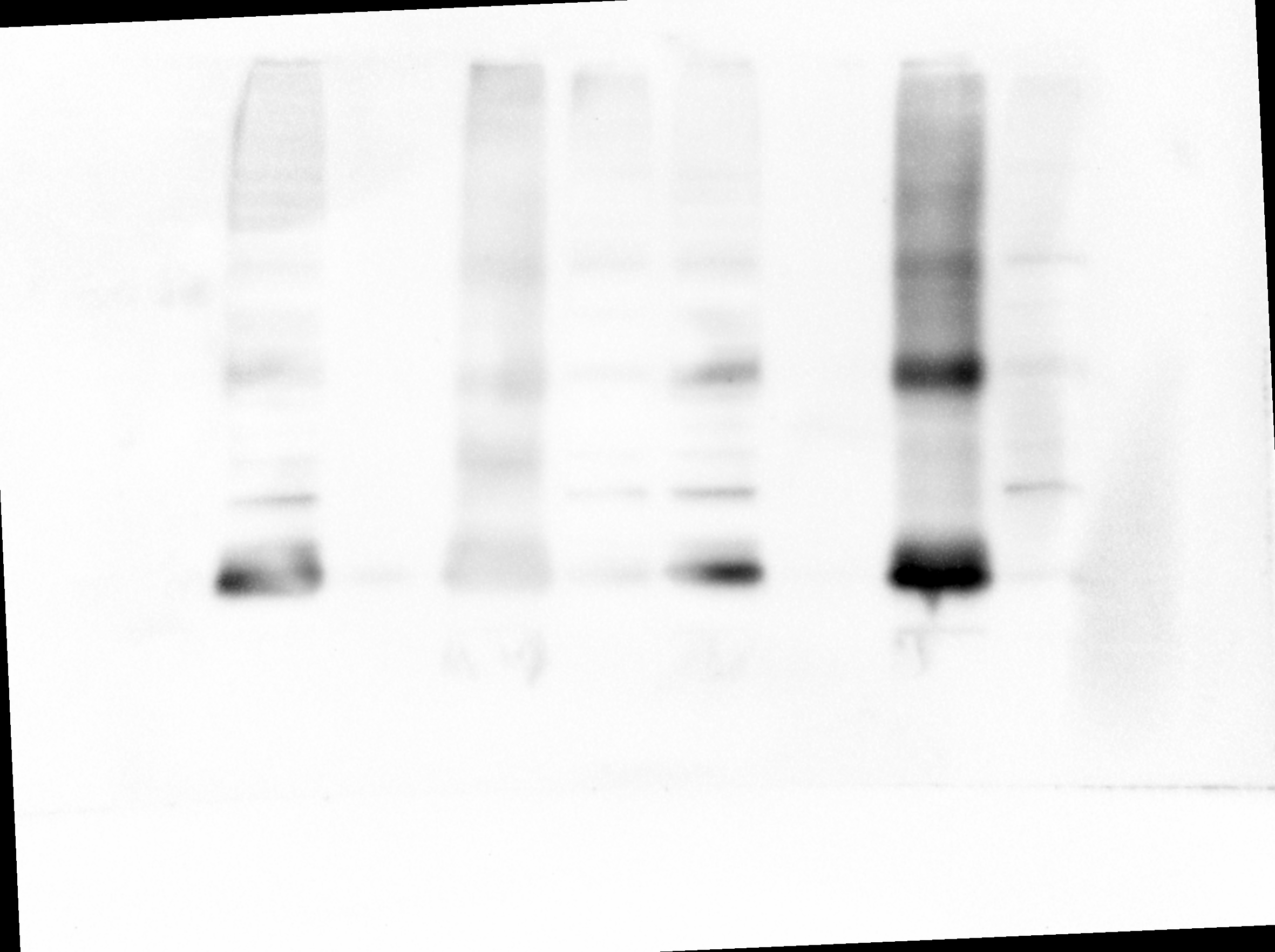

Supplement: Figure 6—source data 4. [file elife-75804-fig6-data4.zip › Figure 6 - Source data 19.tif]

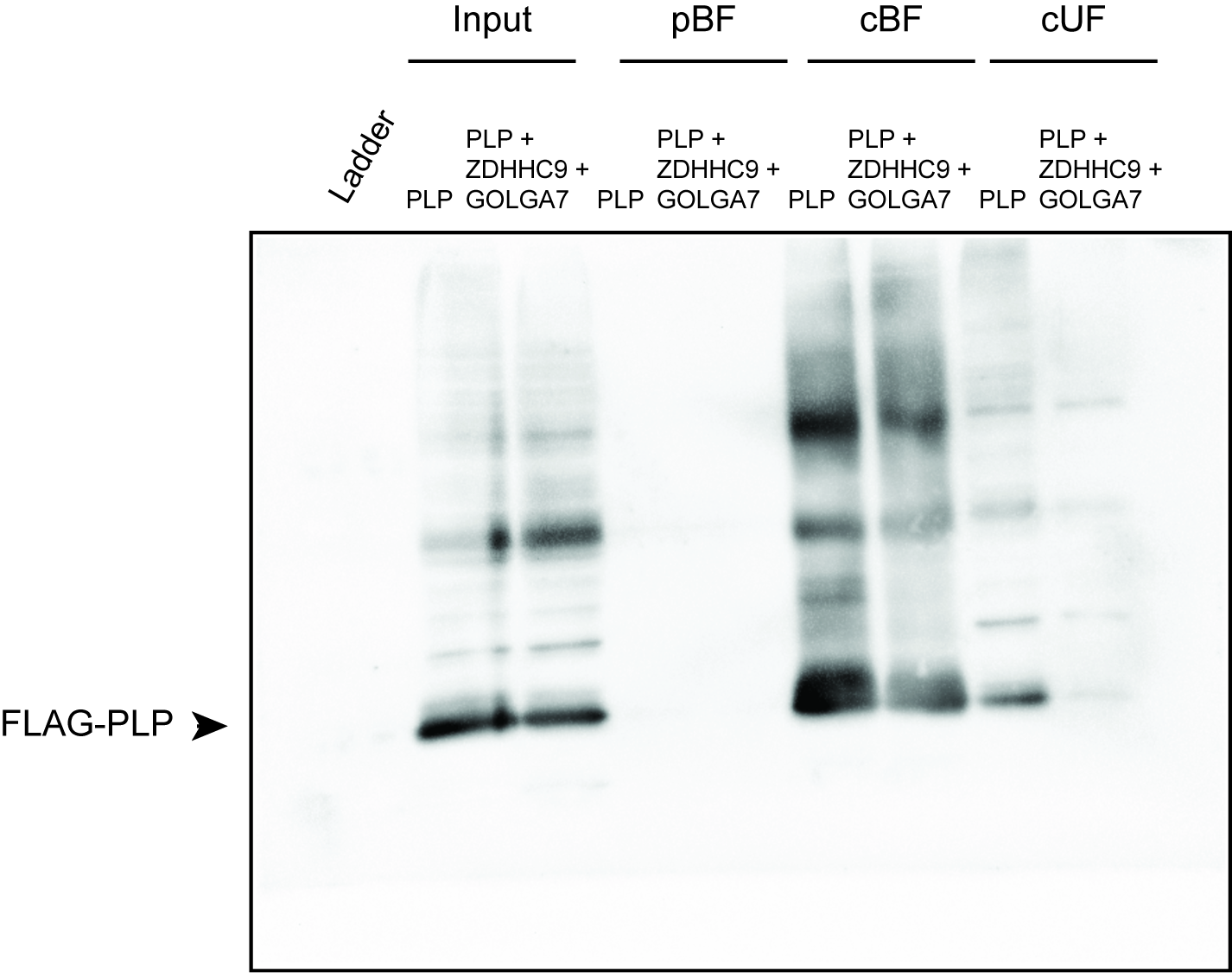

Supplement: Figure 6—source data 4. [file elife-75804-fig6-data4.zip › Figure 6 - Source data 20.tif]

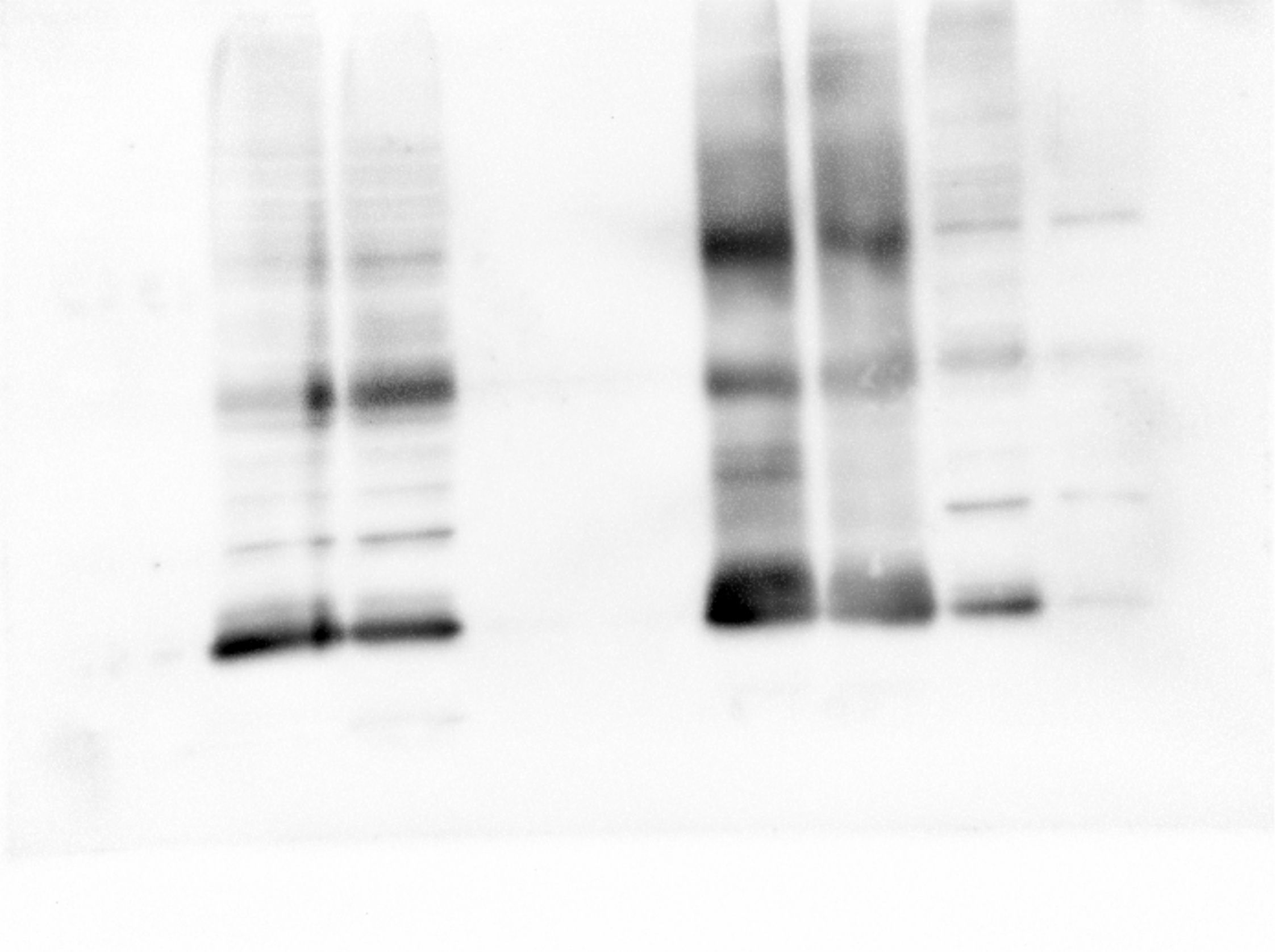

Supplement: Figure 6—source data 4. [file elife-75804-fig6-data4.zip › Figure 6 - Source data 21.tif]

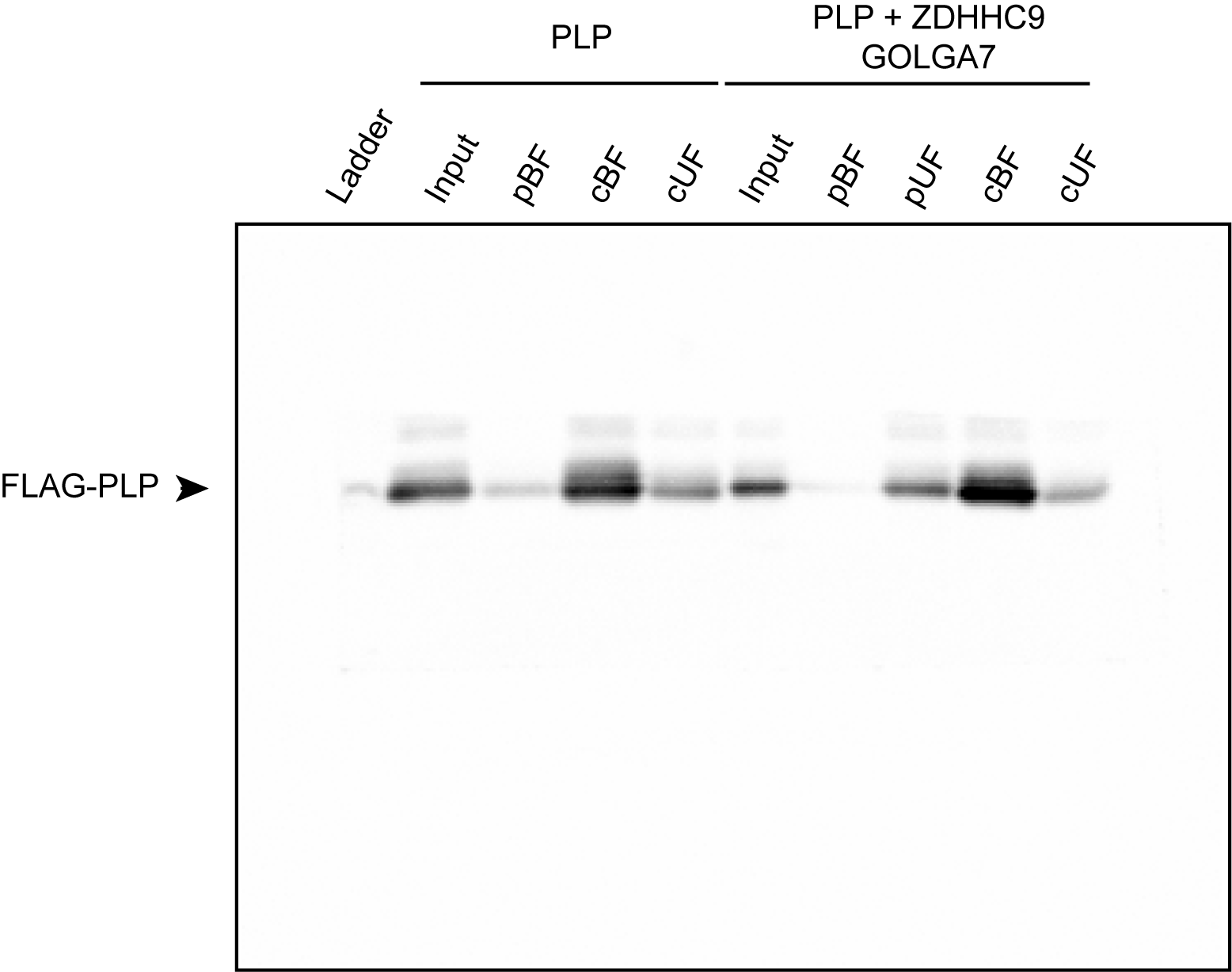

Supplement: Figure 6—source data 4. [file elife-75804-fig6-data4.zip › Figure 6 - Source data 22.tif]

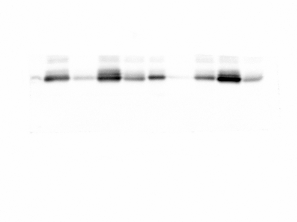

Supplement: Figure 6—source data 4. [file elife-75804-fig6-data4.zip › Figure 6 - Source data 23.tif]

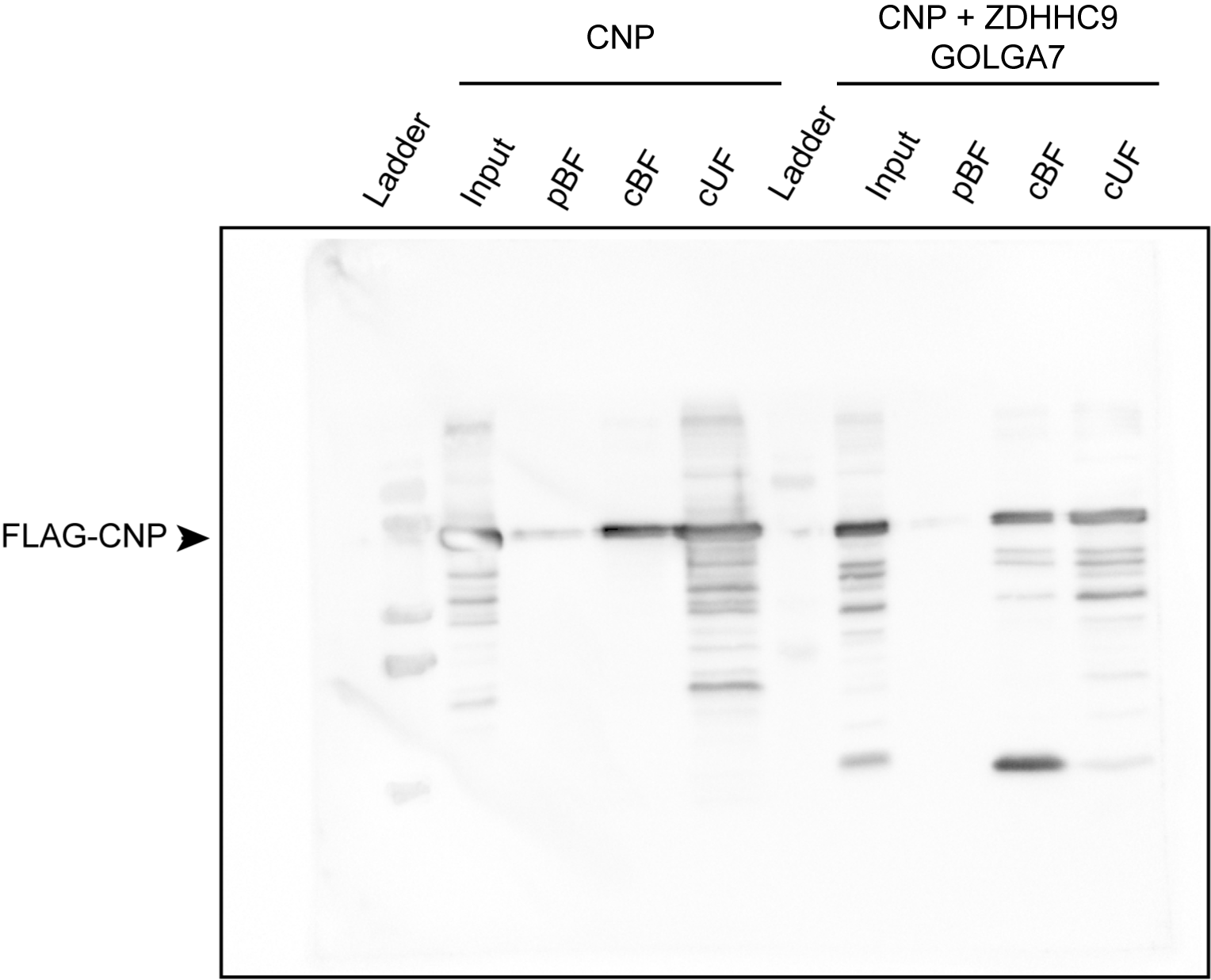

Supplement: Figure 6—source data 4. [file elife-75804-fig6-data4.zip › Figure 6 - Source data 24.tif]

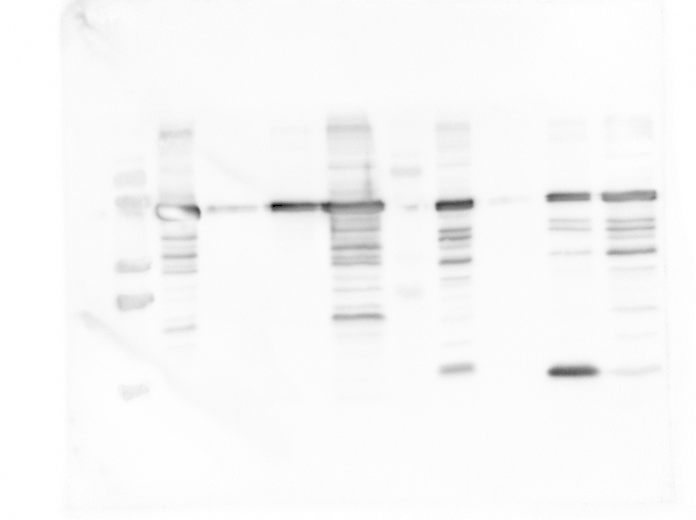

Supplement: Figure 6—source data 4. [file elife-75804-fig6-data4.zip › Figure 6 - Source data 25.tif]

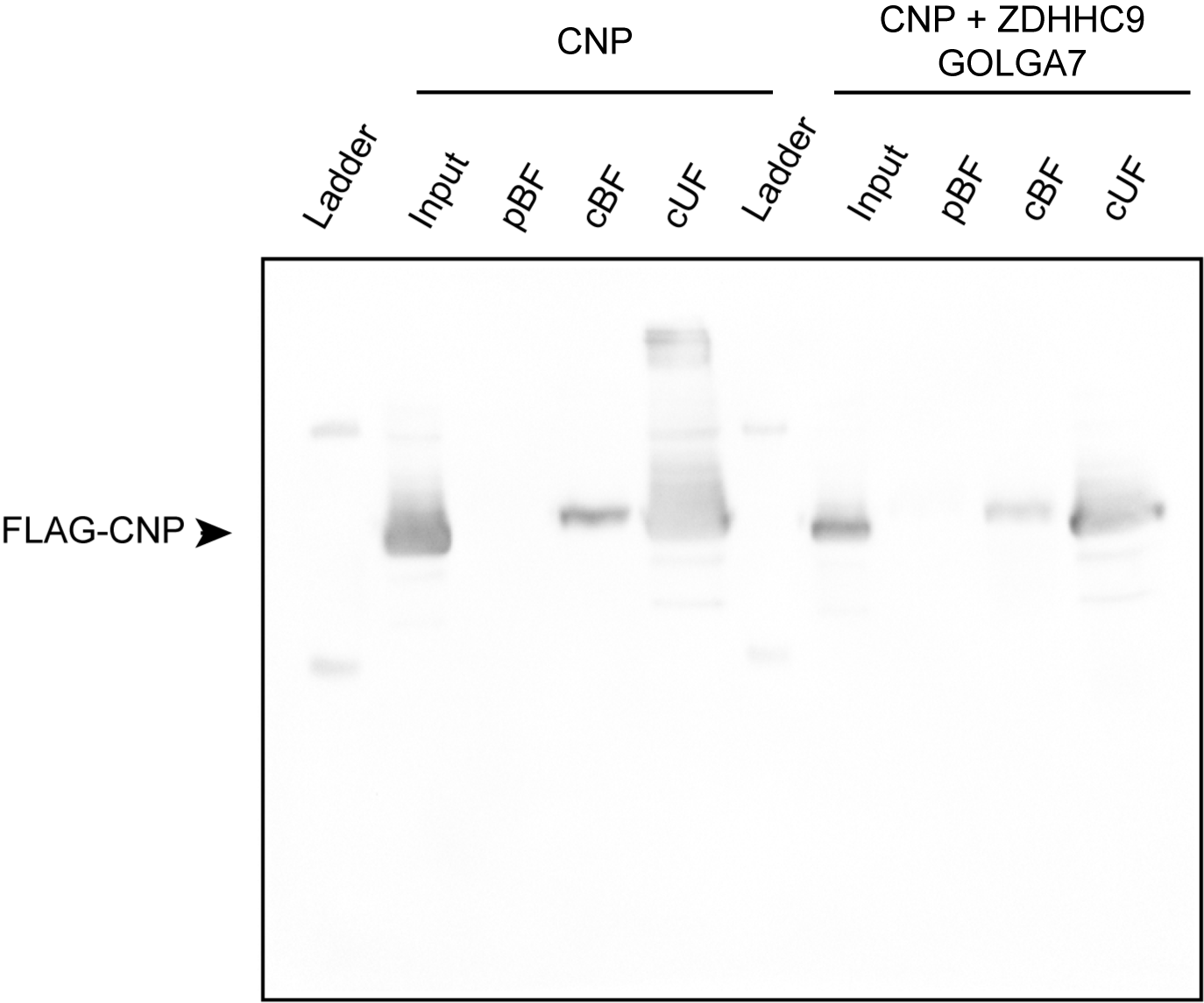

Supplement: Figure 6—source data 4. [file elife-75804-fig6-data4.zip › Figure 6 - Source data 26.tif]

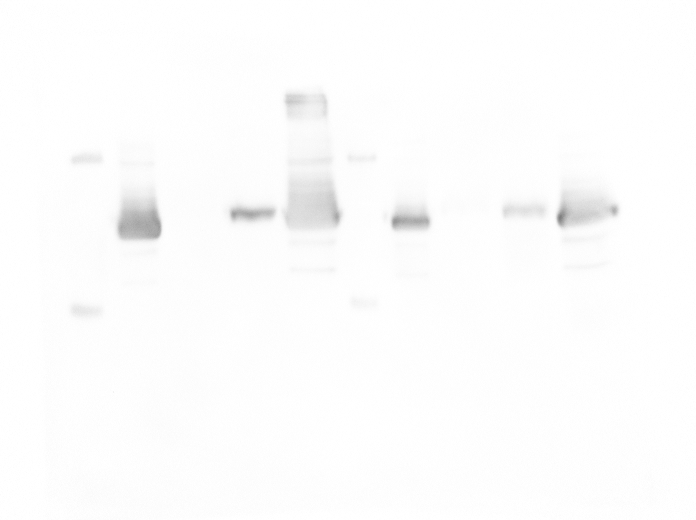

Supplement: Figure 6—source data 4. [file elife-75804-fig6-data4.zip › Figure 6 - Source data 27.tif]

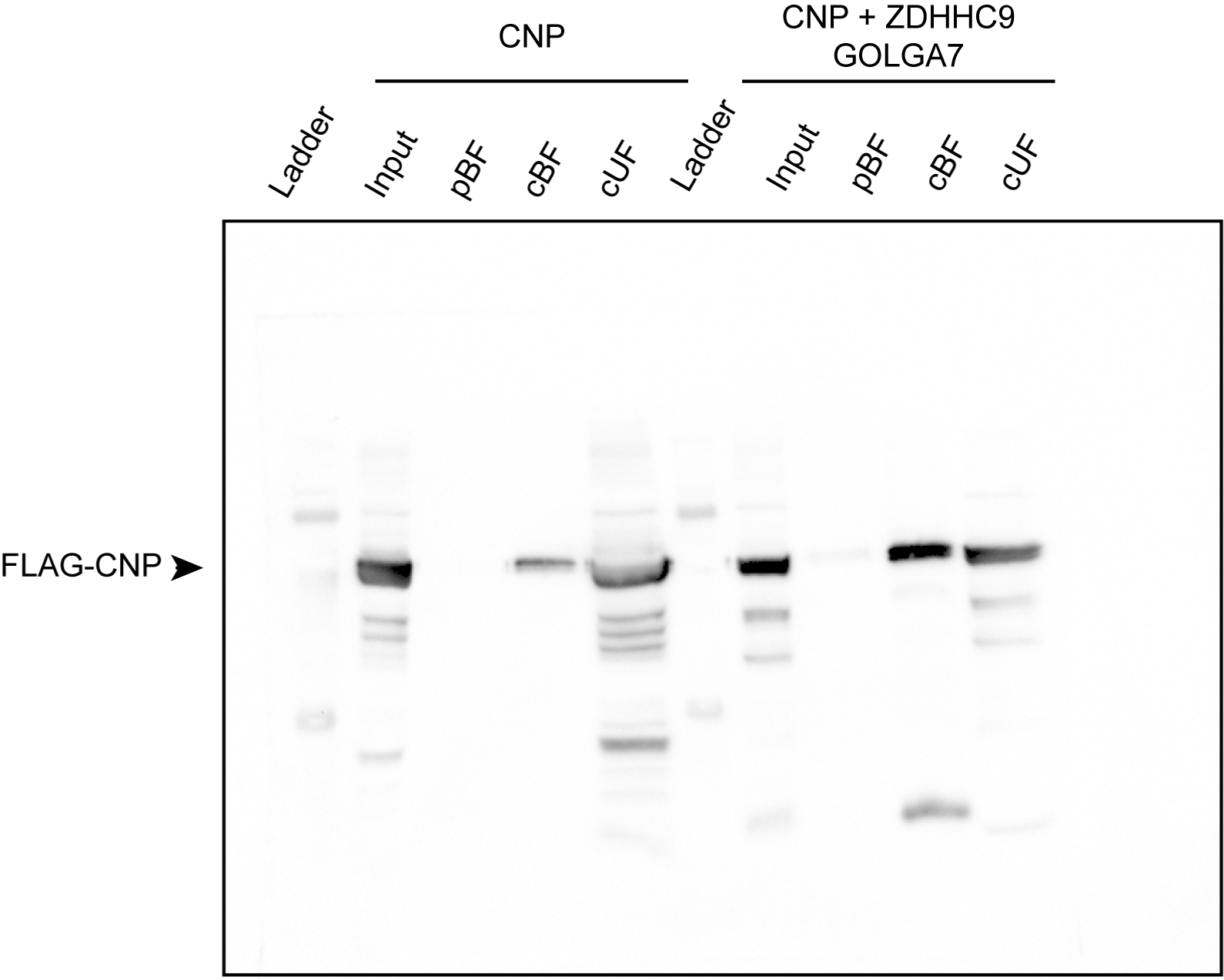

Supplement: Figure 6—source data 4. [file elife-75804-fig6-data4.zip › Figure 6 - Source data 28.tif]

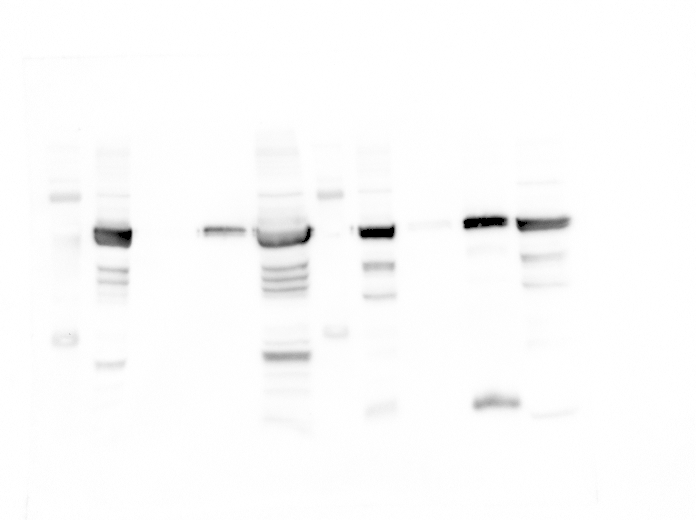

Supplement: Figure 6—source data 4. [file elife-75804-fig6-data4.zip › Figure 6 - Source data 29.tif]

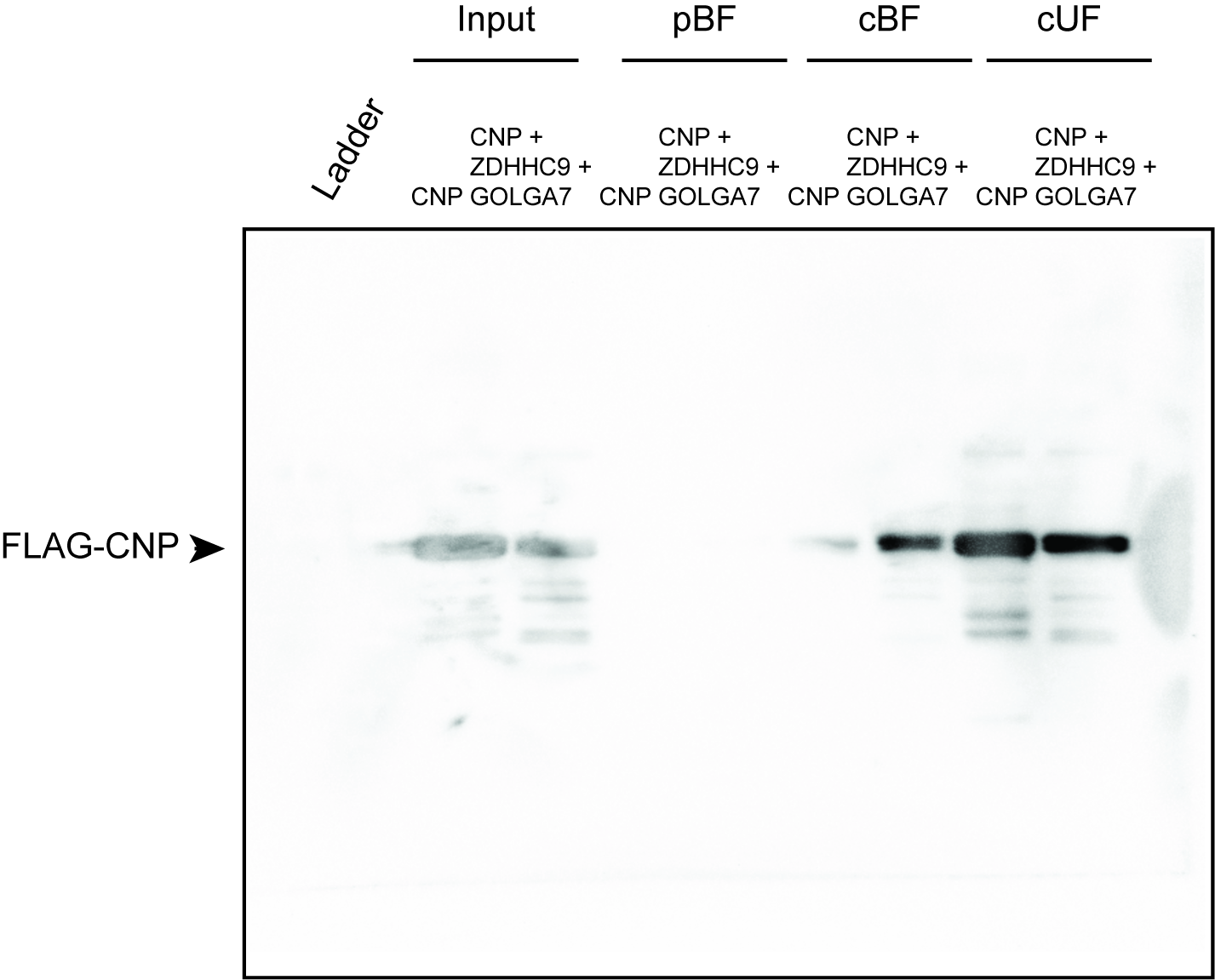

Supplement: Figure 6—source data 4. [file elife-75804-fig6-data4.zip › Figure 6 - Source data 30.tif]

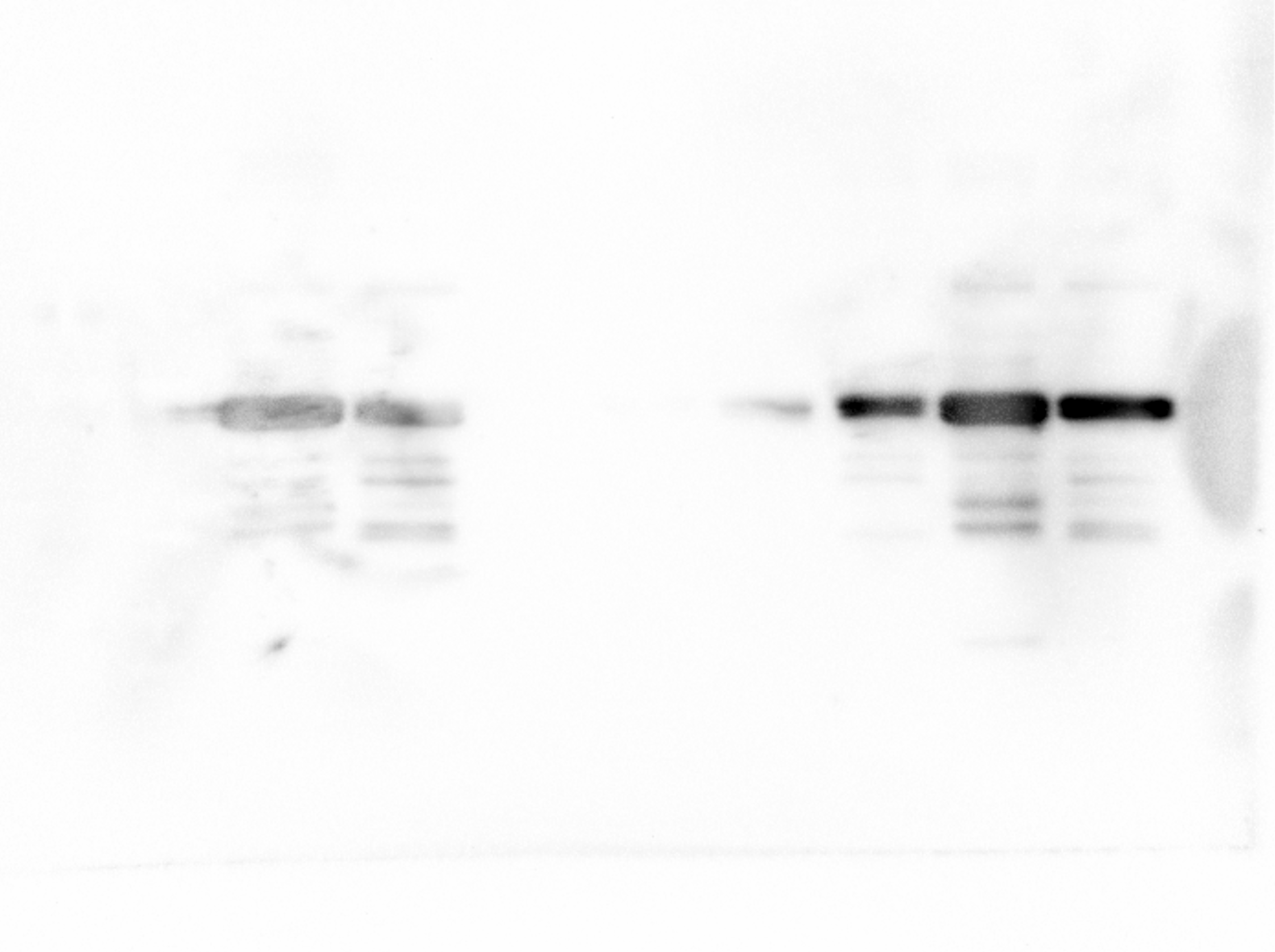

Supplement: Figure 6—source data 4. [file elife-75804-fig6-data4.zip › Figure 6 - Source data 31.tif]

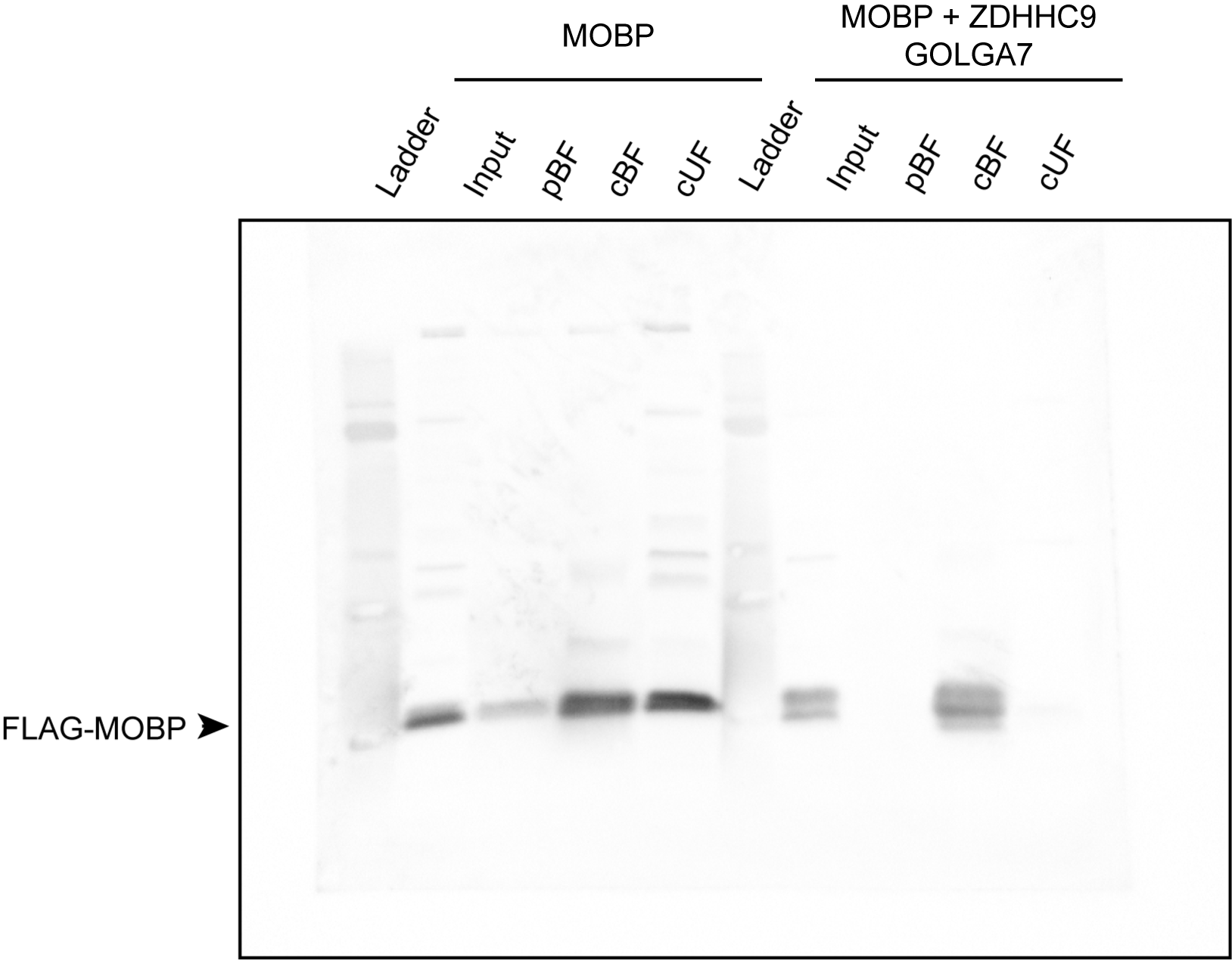

Supplement: Figure 6—source data 4. [file elife-75804-fig6-data4.zip › Figure 6 - Source data 4.tif]

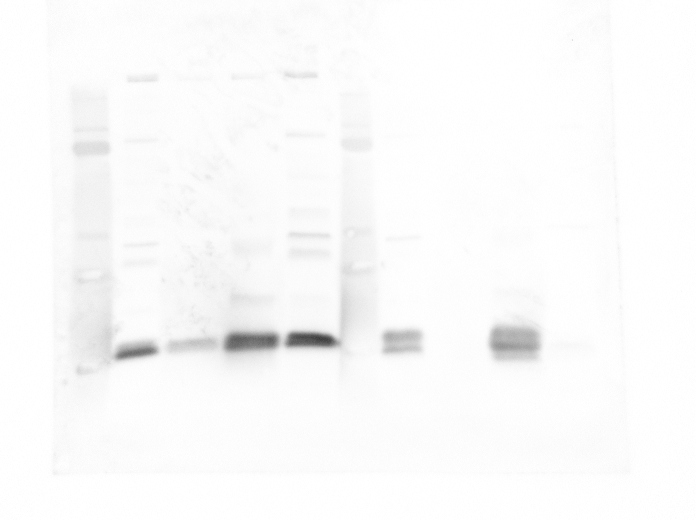

Supplement: Figure 6—source data 4. [file elife-75804-fig6-data4.zip › Figure 6 - Source data 5.tif]

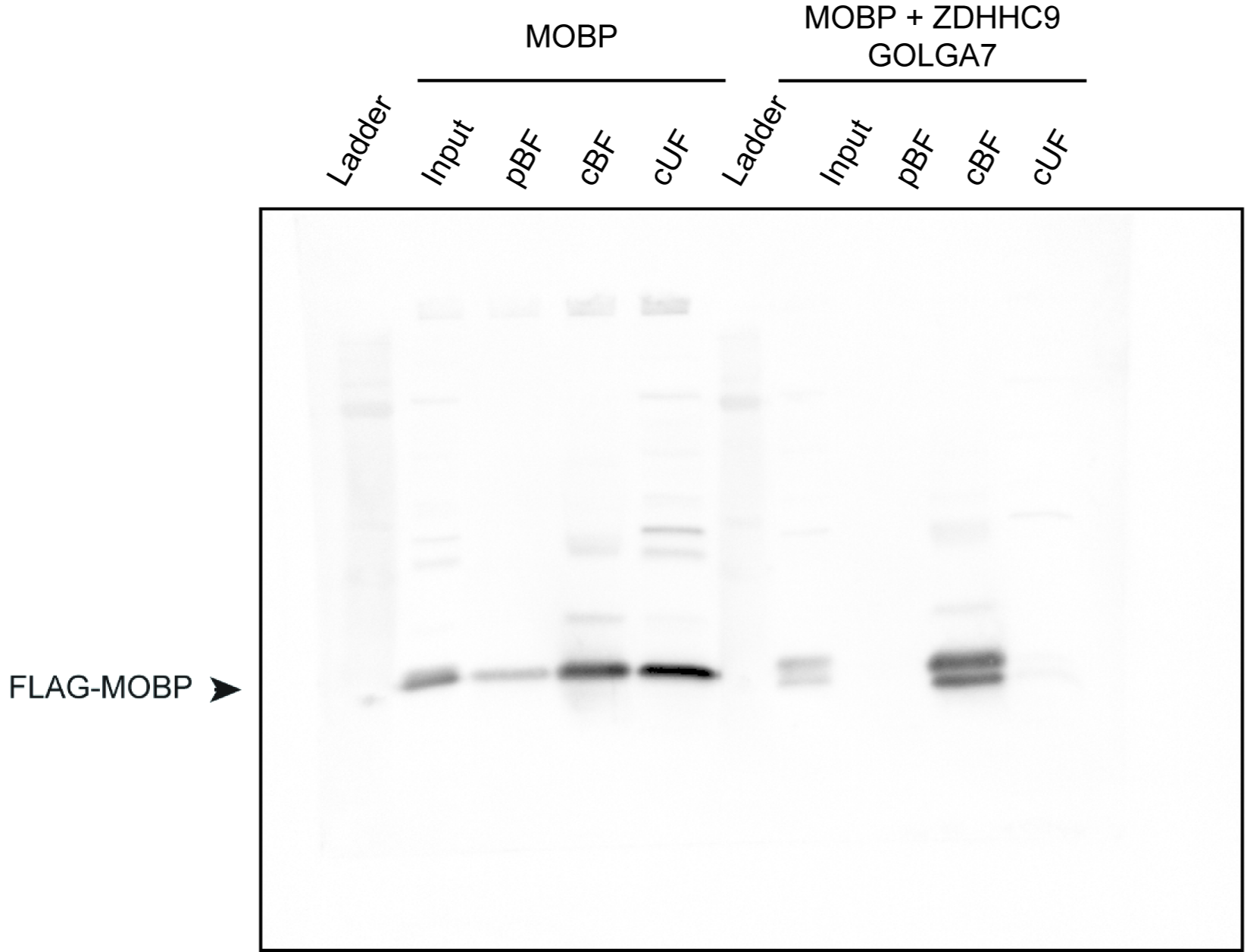

Supplement: Figure 6—source data 4. [file elife-75804-fig6-data4.zip › Figure 6 - Source data 6.tif]

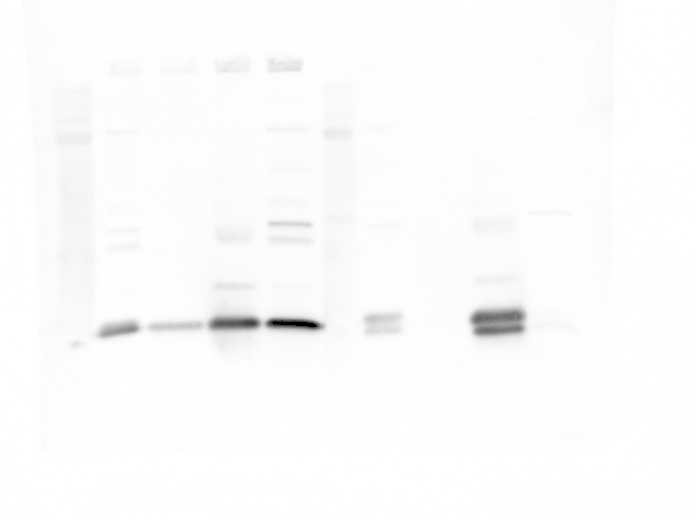

Supplement: Figure 6—source data 4. [file elife-75804-fig6-data4.zip › Figure 6 - Source data 7.tif]

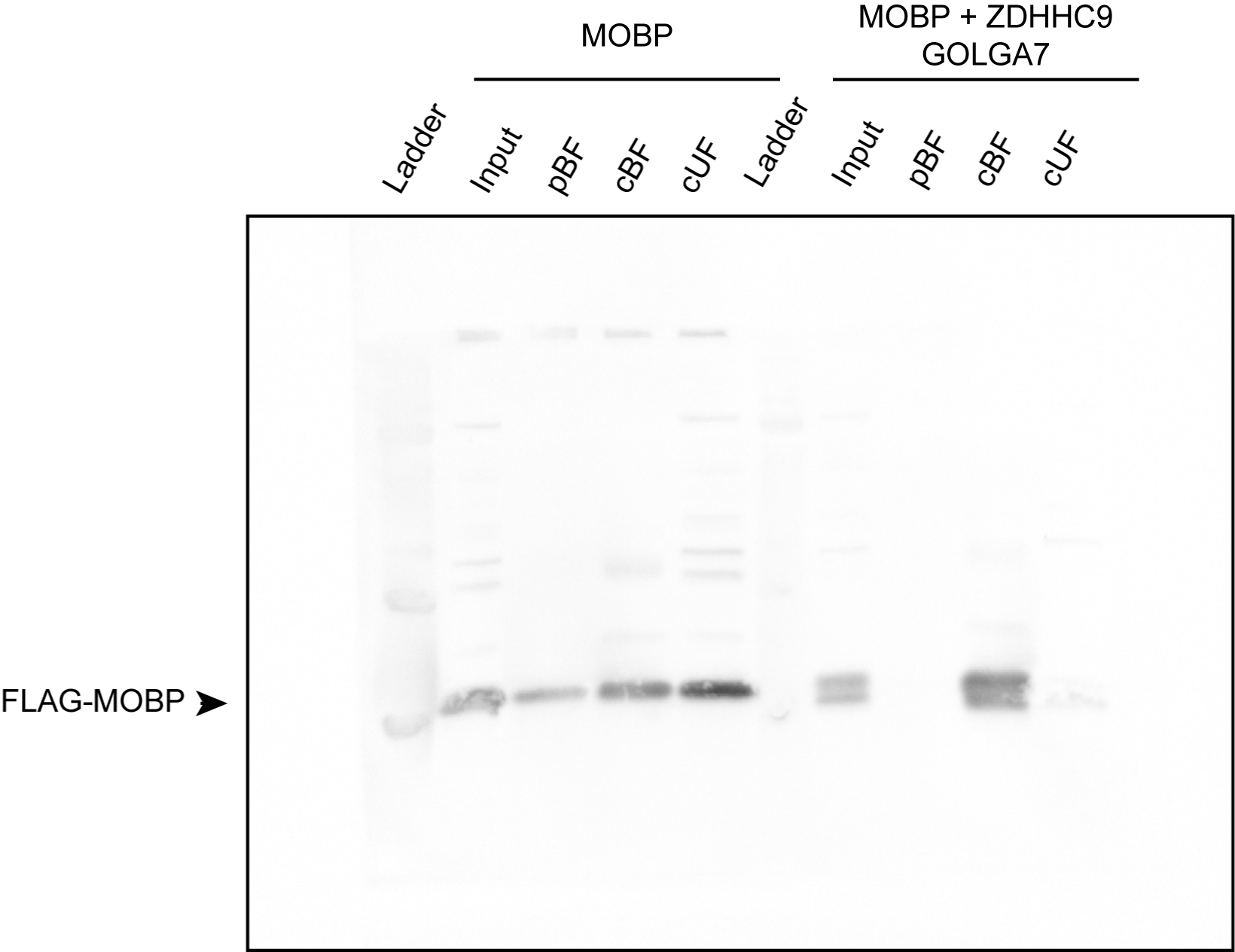

Supplement: Figure 6—source data 4. [file elife-75804-fig6-data4.zip › Figure 6 - Source data 8.tif]

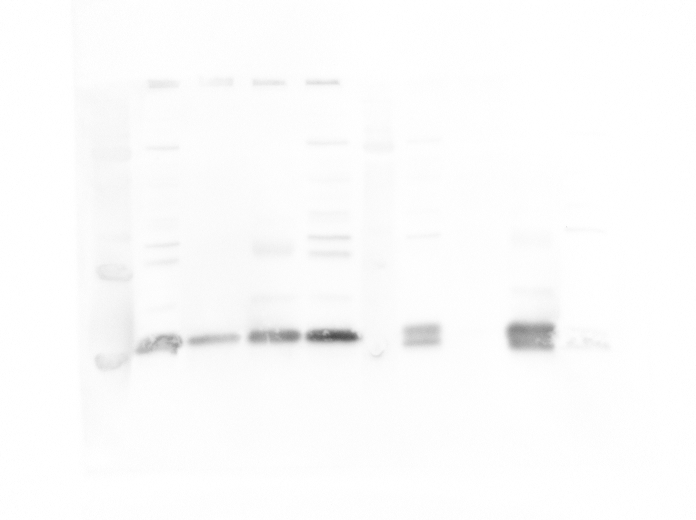

Supplement: Figure 6—source data 4. [file elife-75804-fig6-data4.zip › Figure 6 - Source data 9.tif]

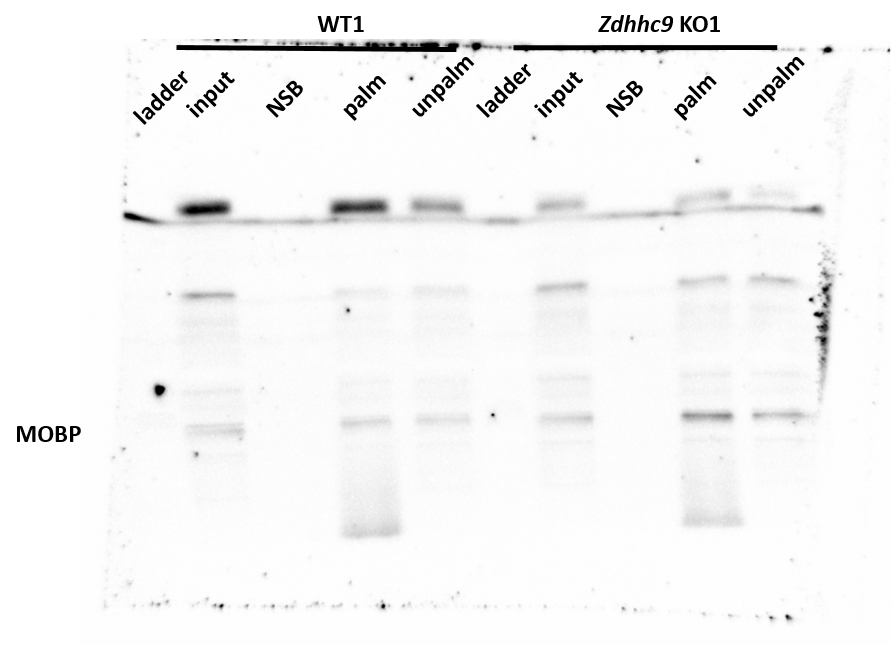

Supplement: Figure 6—figure supplement 2—source data 1. [file elife-75804-fig6-figsupp2-data1.zip › Figure 6 - Figure supplement 2 -source data 10.tif]

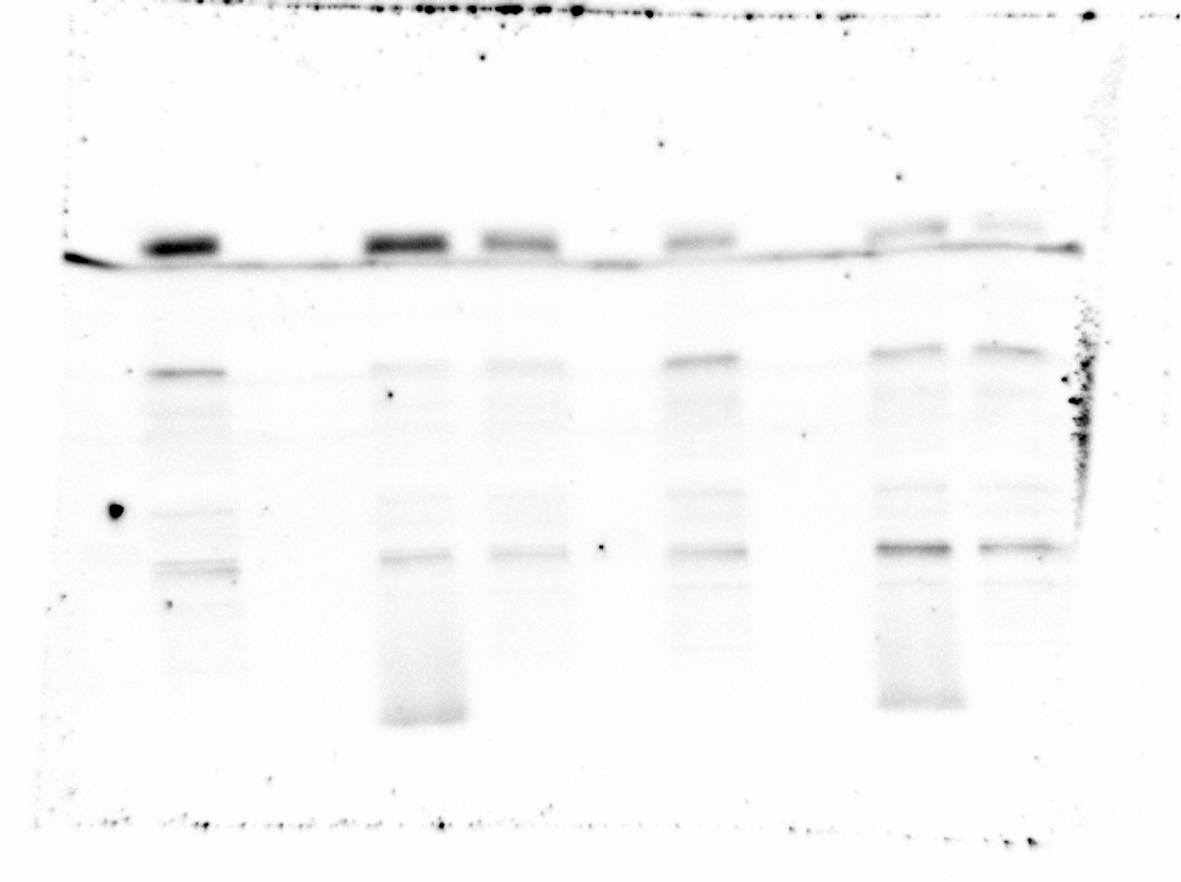

Supplement: Figure 6—figure supplement 2—source data 1. [file elife-75804-fig6-figsupp2-data1.zip › Figure 6 - Figure supplement 2 -source data 11.tif]

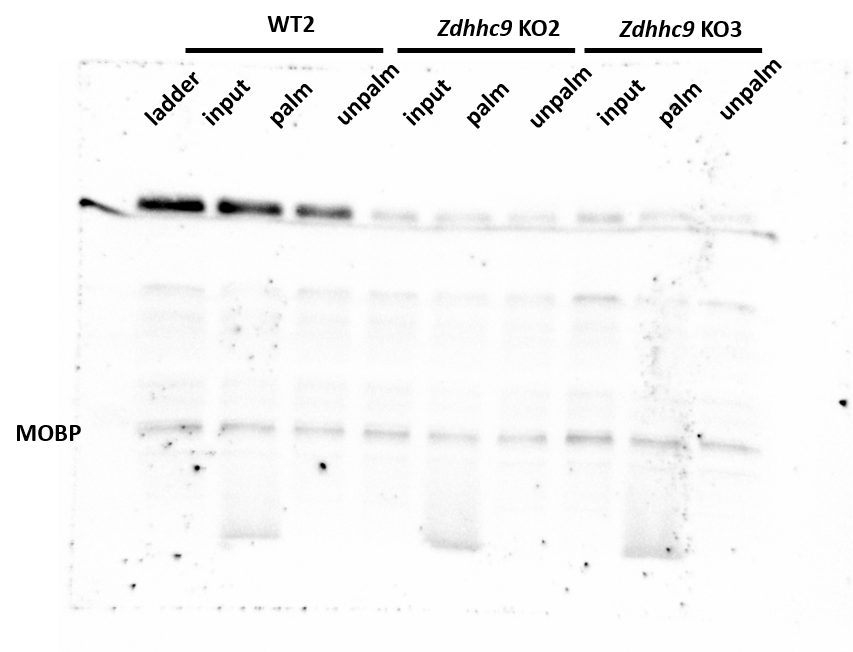

Supplement: Figure 6—figure supplement 2—source data 1. [file elife-75804-fig6-figsupp2-data1.zip › Figure 6 - Figure supplement 2 -source data 12.tif]

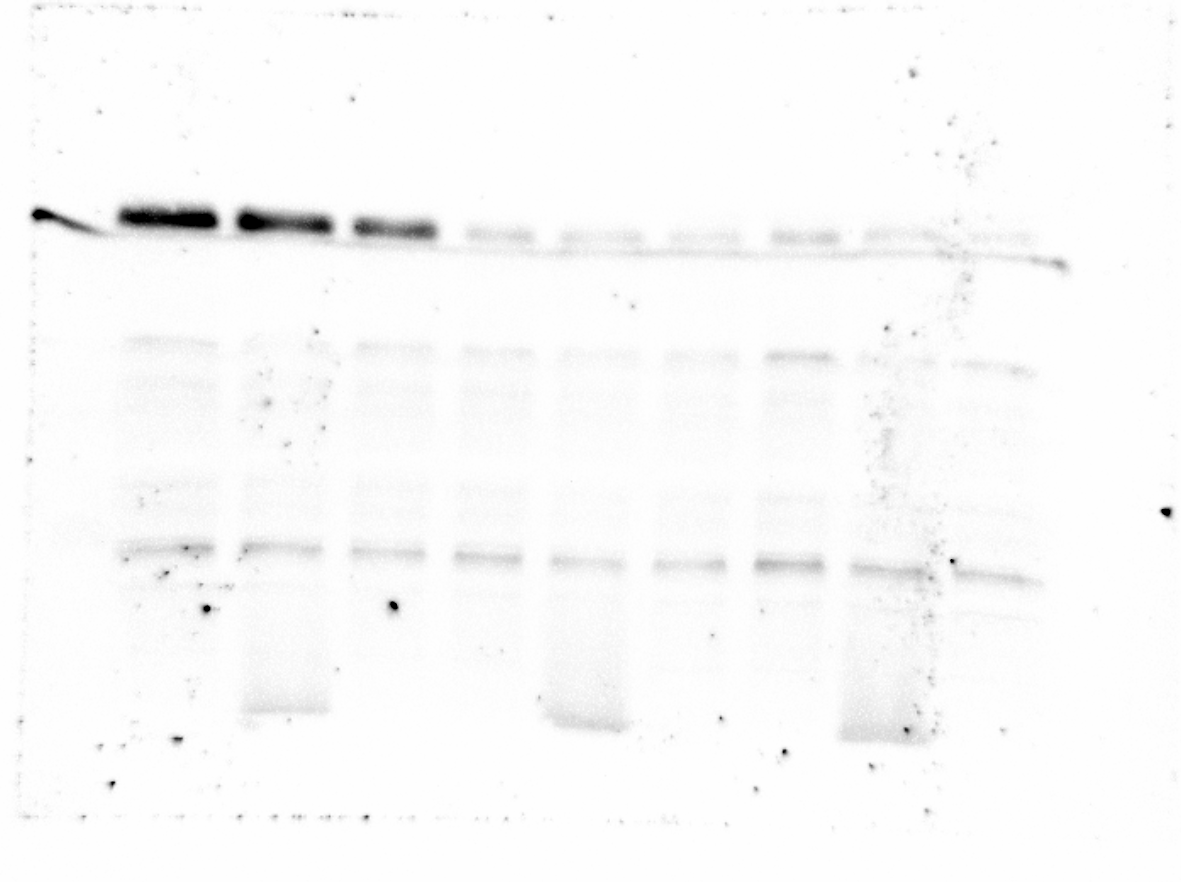

Supplement: Figure 6—figure supplement 2—source data 1. [file elife-75804-fig6-figsupp2-data1.zip › Figure 6 - Figure supplement 2 -source data 13.tif]

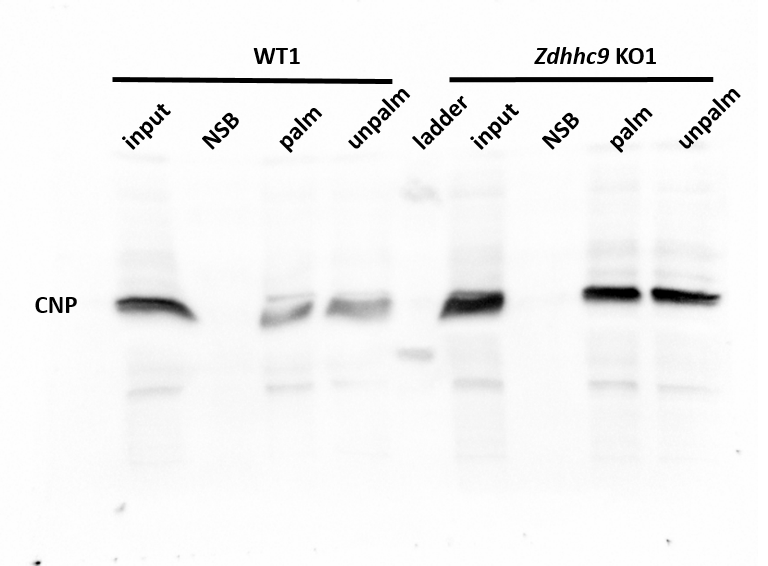

Supplement: Figure 6—figure supplement 2—source data 1. [file elife-75804-fig6-figsupp2-data1.zip › Figure 6 - Figure supplement 2 -source data 2.tif]

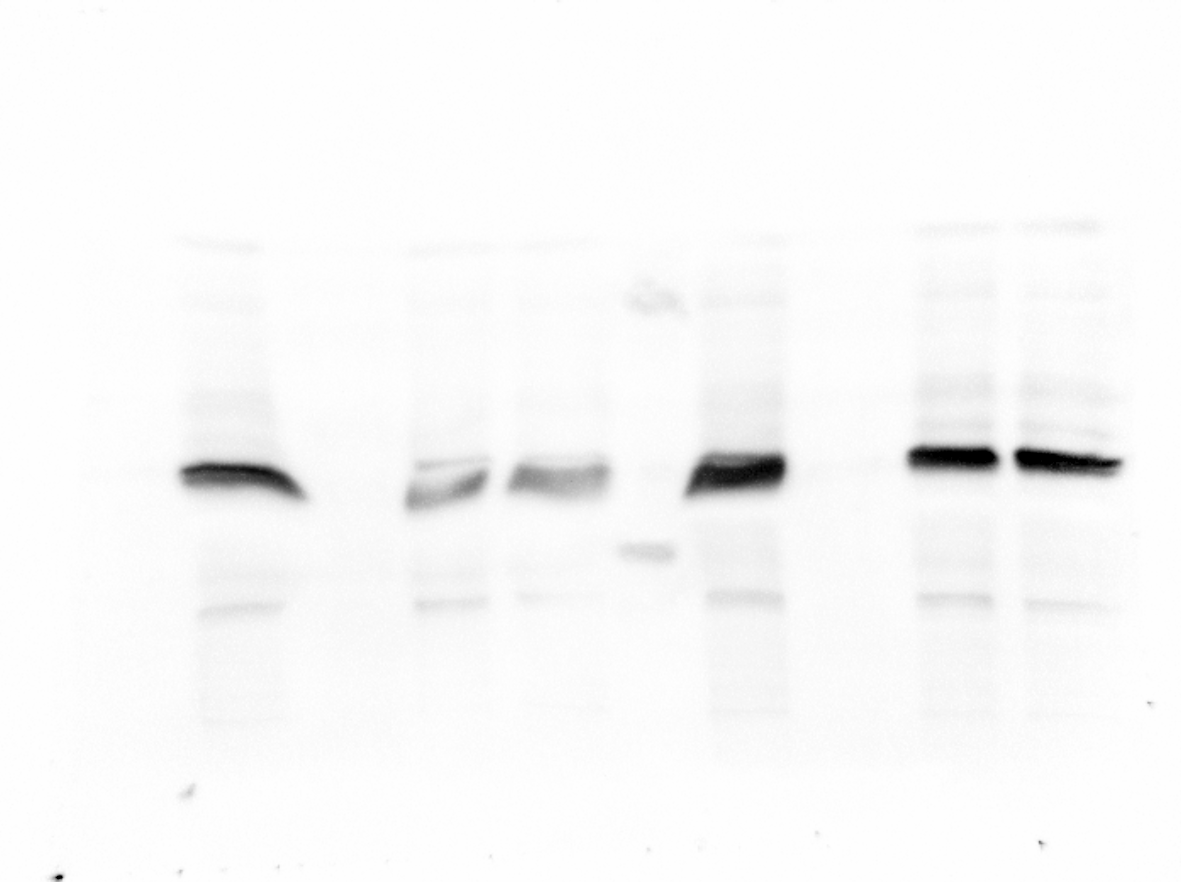

Supplement: Figure 6—figure supplement 2—source data 1. [file elife-75804-fig6-figsupp2-data1.zip › Figure 6 - Figure supplement 2 -source data 3.tif]

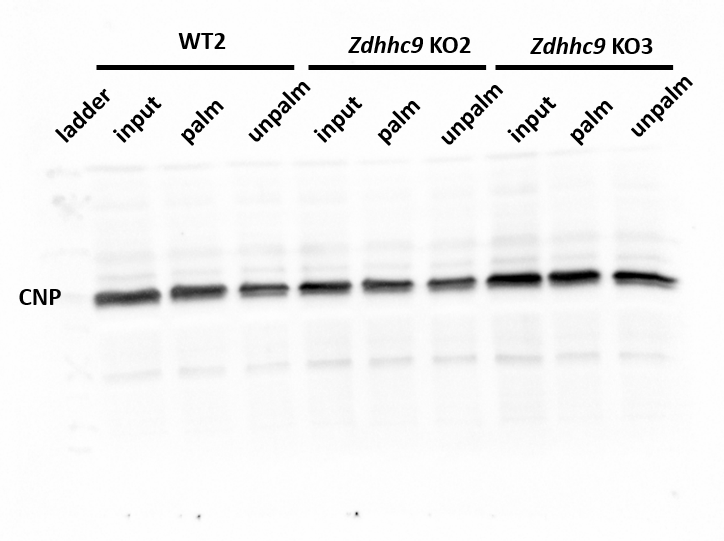

Supplement: Figure 6—figure supplement 2—source data 1. [file elife-75804-fig6-figsupp2-data1.zip › Figure 6 - Figure supplement 2 -source data 4.tif]

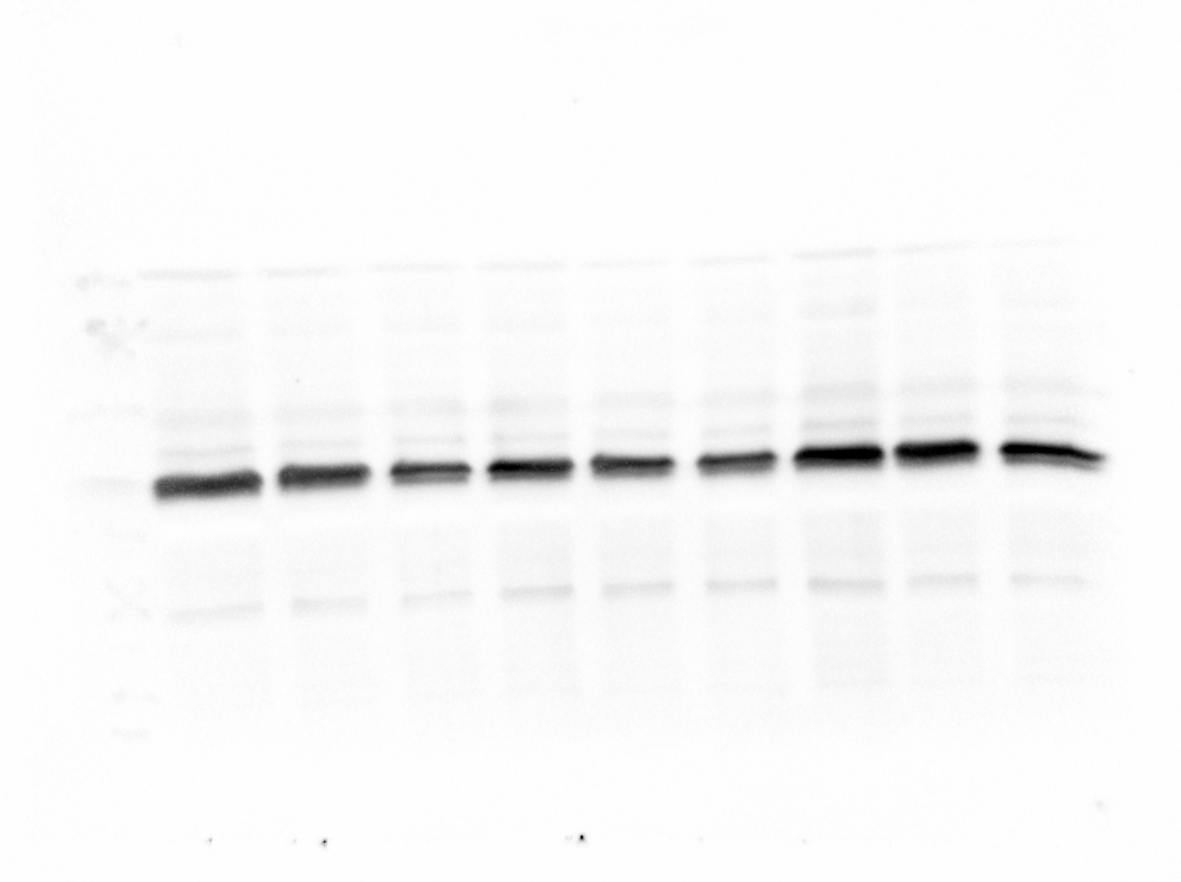

Supplement: Figure 6—figure supplement 2—source data 1. [file elife-75804-fig6-figsupp2-data1.zip › Figure 6 - Figure supplement 2 -source data 5.tif]

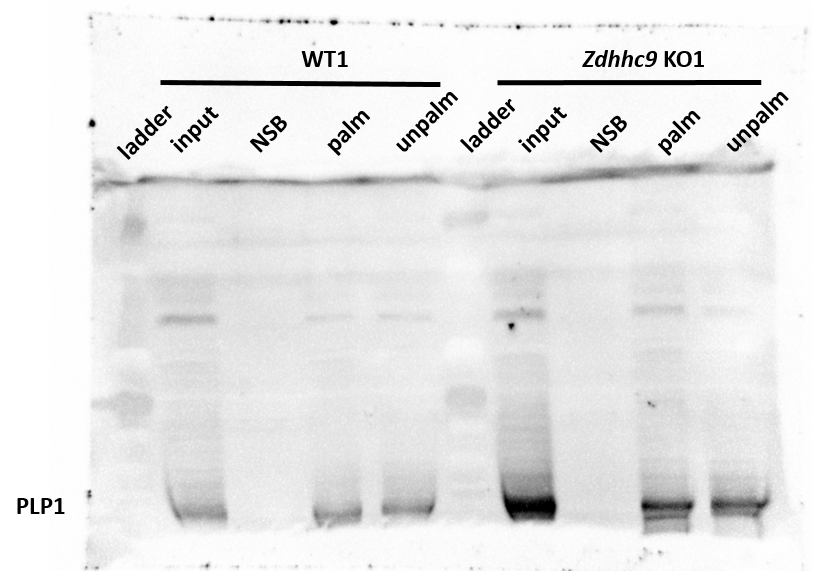

Supplement: Figure 6—figure supplement 2—source data 1. [file elife-75804-fig6-figsupp2-data1.zip › Figure 6 - Figure supplement 2 -source data 6.tif]

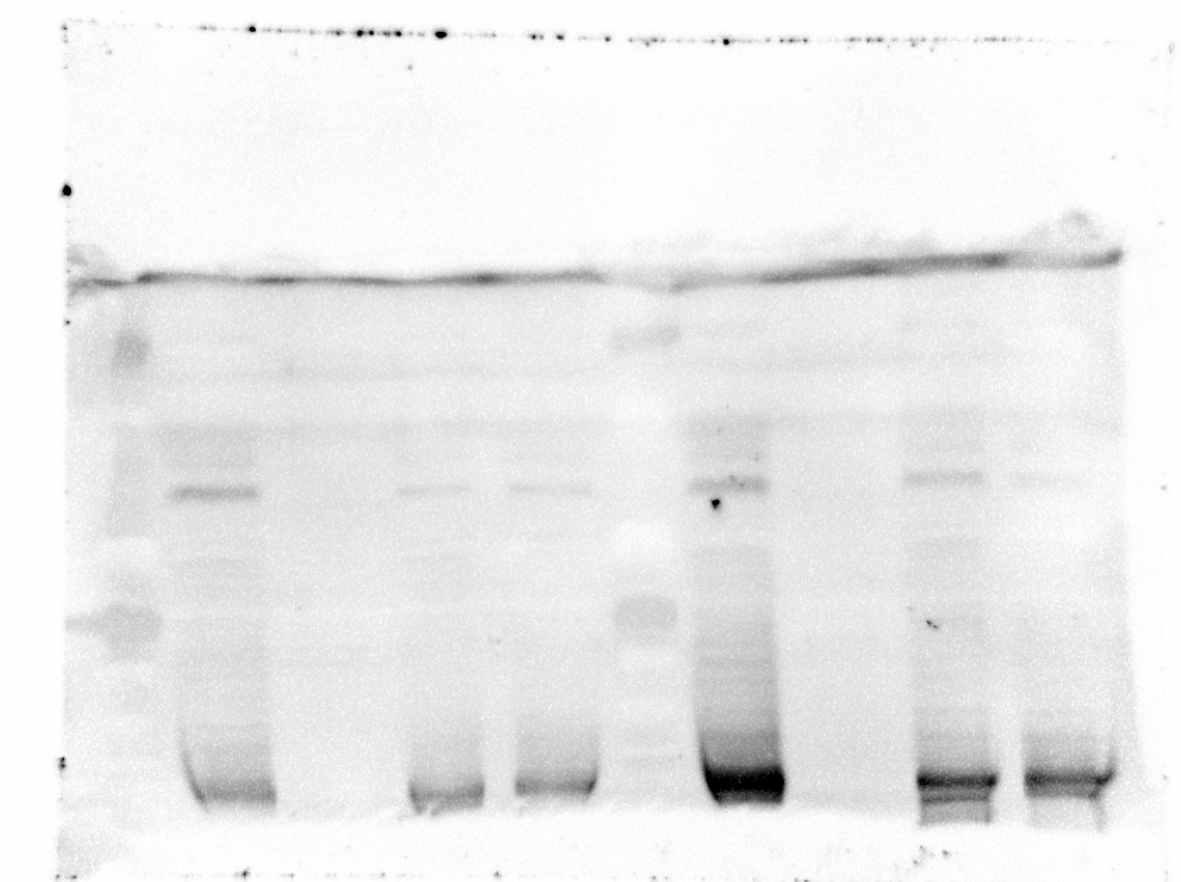

Supplement: Figure 6—figure supplement 2—source data 1. [file elife-75804-fig6-figsupp2-data1.zip › Figure 6 - Figure supplement 2 -source data 7.tif]

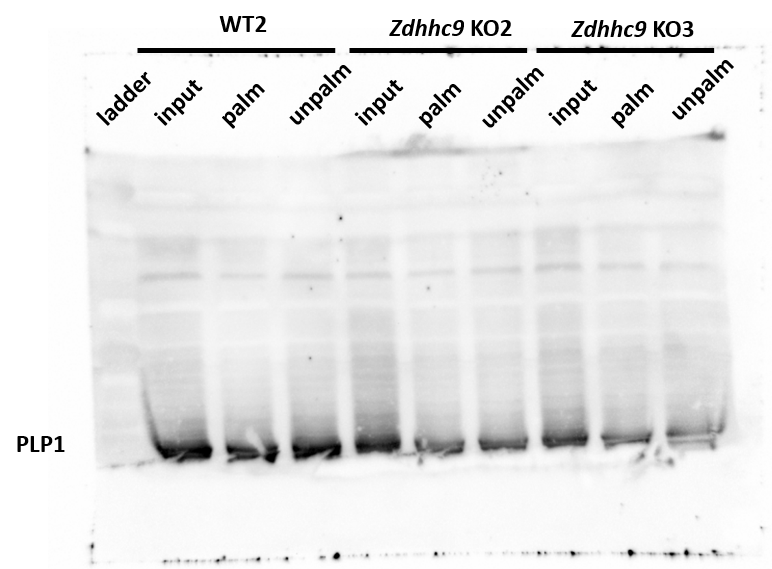

Supplement: Figure 6—figure supplement 2—source data 1. [file elife-75804-fig6-figsupp2-data1.zip › Figure 6 - Figure supplement 2 -source data 8.tif]

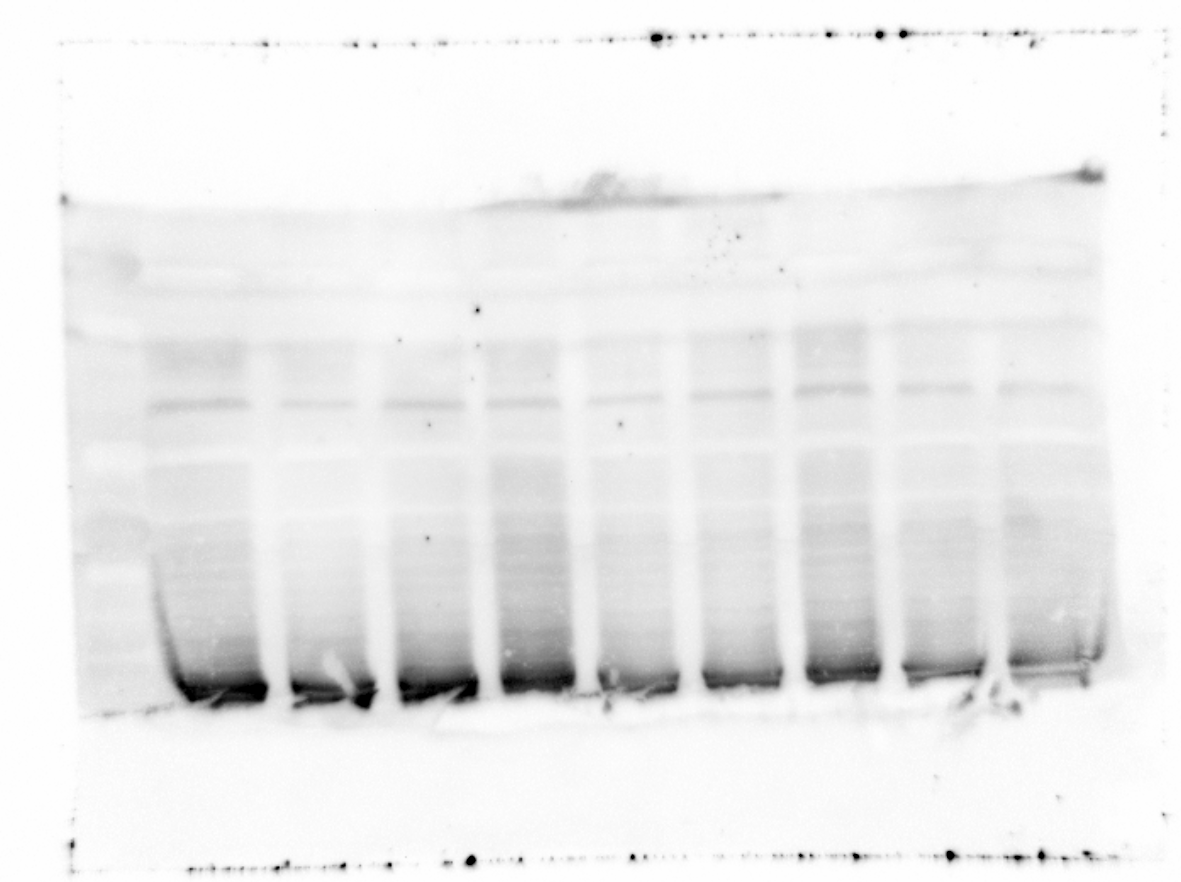

Supplement: Figure 6—figure supplement 2—source data 1. [file elife-75804-fig6-figsupp2-data1.zip › Figure 6 - Figure supplement 2 -source data 9.tif]
